# Comprehensive transcriptomic analysis of heat shock proteins in the molecular subtypes of human breast cancer

**DOI:** 10.1186/s12885-018-4621-1

**Published:** 2018-06-28

**Authors:** Felipe C. M. Zoppino, Martin E. Guerrero-Gimenez, Gisela N. Castro, Daniel R. Ciocca

**Affiliations:** Laboratory of Oncology, Institute of Medicine and Experimental Biology of Cuyo (IMBECU), National Scientific and Technical Research Council (CONICET), Av. Dr. Ruiz Leal s/n, Parque General San Martín, 5500 Mendoza, Argentina

**Keywords:** Breast cancer, Heat shock proteins, Differential gene expression, Molecular subtypes, Survival, HSP-Clusts

## Abstract

**Background:**

Heat Shock Proteins (HSPs), a family of genes with key roles in proteostasis, have been extensively associated with cancer behaviour. However, the HSP family is quite large and many of its members have not been investigated in breast cancer (BRCA), particularly in relation with the current molecular BRCA classification. In this work, we performed a comprehensive transcriptomic study of the HSP gene family in BRCA patients from both The Cancer Genome Atlas (TCGA) and the Molecular Taxonomy of Breast Cancer International Consortium (METABRIC) cohorts discriminating the BRCA intrinsic molecular subtypes.

**Methods:**

We examined gene expression levels of 1097 BRCA tissue samples retrieved from TCGA and 1981 samples of METABRIC, focusing mainly on the HSP family (95 genes). Data were stratified according to the PAM50 gene expression (Luminal A, Luminal B, HER2, Basal, and Normal-like). Transcriptomic analyses include several statistical approaches: differential gene expression, hierarchical clustering and survival analysis.

**Results:**

Of the 20,531 analysed genes we found that in BRCA almost 30% presented deregulated expression (19% upregulated and 10% downregulated), while of the HSP family 25% appeared deregulated (14% upregulated and 11% downregulated) (|fold change| > 2 comparing BRCA with normal breast tissues). The study revealed the existence of shared HSP genes deregulated in all subtypes of BRCA while other HSPs were deregulated in specific subtypes. Many members of the Chaperonin subfamily were found upregulated while three members (BBS10, BBS12 and CCTB6) were found downregulated. HSPC subfamily had moderate increments of transcripts levels. Various genes of the HSP70 subfamily were upregulated; meanwhile, HSPA12A and HSPA12B appeared strongly downregulated. The strongest downregulation was observed in several HSPB members except for HSPB1. DNAJ members showed heterogeneous expression pattern. We found that 23 HSP genes correlated with overall survival and three HSP-based transcriptional profiles with impact on disease outcome were recognized.

**Conclusions:**

We identified shared and specific HSP genes deregulated in BRCA subtypes. This study allowed the recognition of HSP genes not previously associated with BRCA and/or any cancer type, and the identification of three clinically relevant clusters based on HSPs expression patterns with influence on overall survival.

**Electronic supplementary material:**

The online version of this article (10.1186/s12885-018-4621-1) contains supplementary material, which is available to authorized users.

## Background

In worldwide terms, breast cancer (BRCA) has the second annual incidence (1,670,000 cases) and the fifth mortality rate (522,000 deaths associated) of overall cancers [1]. Classifications of BRCA have been performed according to clinical features, histological characteristics, and presence of steroid and/or growth factor receptors. PAM50 gene expression assay allows the molecular classification of BRCA based on the expression levels of fifty genes and sorts BRCA into five intrinsic subtypes: Luminal A, Luminal B, HER2-enriched (HER2), Basal-like (Basal) and Normal-like (Normal). This classification highly correlates with BRCA biological behaviour and has clinical use due to its prognostic significance [2, 3]. Heat Shock Proteins (HSPs) are ubiquitous in living organisms and their expression is rapidly regulated by stress. Historically they were recognized as proteins induced by heat, although it is now known that various types of physiological and/or pathological stresses regulate their expression [4]. HSP systems are involved in protein quality control [5], degradation pathways (ubiquitin-proteasome system, endoplasmic reticulum associated degradation, autophagy), and regulation of apoptosis [5, 6]. The HSPs belong to a family of evolutionarily conserved genes that includes 95 genes divided into five subfamilies: 1) type I chaperonins (HSP10 and HSP60), BBs chaperonins, and type II chaperonins (CCT genes) which are grouped under the Chaperonin subfamily (CHAP); 2) HSP70 (HSPA) and large HSP 100–110 kDa (which are all included in the HSP70 family); 3) small HSP 12–43 kDa (HSPB); 4) HSP90 (HSPC); and 5) HSP40 (DNAJ) [7]. The HSPs related systems can be disturbed during oncogenesis allowing malignant transformation and/or facilitating rapid somatic evolution; they have been studied in a wide variety of cancers, presenting different pro-tumour (stimulating tumour growth and metastasis) or anti-tumour actions [4, 8]. Currently, HSPs are emerging as molecular targets in cancer therapy through the interference of their diversity of functions in cancer cells by different approaches. In fact, there are clinical trials for various cancers, including BRCA, using HSP-inhibitor compounds and other HSP-based strategies [9–11]. The information gathered from diverse studies regarding the role of the HSPs in different situations associated with cancer frequently provides contradictory overviews. HSP genes (and encoded proteins) corresponding to HSPA1A/B, HSPB1, DNAJB1 and HSP90AA1 are the most studied; these have been tested in various models (cell culture, biopsies, etc.), nevertheless in the context of BRCA many others HSPs have not been studied yet. Currently, we have not found specific studies of the complete HSP gene family in BRCA integrating the multi-omics platforms available. The participation and implications of HSPs involved in different pathways controlling cell growth, differentiation and apoptosis emphasize the importance for a thorough and comprehensive study of all members of these genes. The purpose of this study is the analysis and integration of clinical and transcriptomic (RNAseq) data of BRCA tumour samples from TCGA and METABRIC databases with emphasis on HSP genes in the five BRCA molecular subtypes. We hypothesize that the results of this investigation will generate relevant knowledge of the HSPs expression landscape, useful in the genomic and clinical characterization of BRCA.

## Methods

### Data analyses

Two independent datasets were used in this study: 1) The “TCGA assembler” v.1.0.3 [12] package was used to programmatically download, from the publicly available TCGA (http://cancergenome.nih.gov/) dataset of mammary adenocarcinoma, level 3 standardized (normalized) and non-standardized (raw counts) mRNA gene expression levels from 1097 tumour samples and 114 normal tissue samples measured using the RNA-Seq technology (RNASeqV2) (May 1, 2015). Available clinical information corresponding to 1085 patients was obtained using the same package and updated with the latest follow-up available. Samples were obtained from patients with initial diagnosis of invasive breast adenocarcinoma undergoing surgical resection and that had no prior treatment for their diseases. Samples were collected between 1988 and 2013, disregarding gender, race, histological type, disease stage or other co-morbidities **(**Additional file 1: Table S1). The tumour sections analysed were required to contain an average of 60% tumour cell nuclei with less than 20% necrosis under TCGA protocol standards. The treatments of patients varied according to the standard of treatment at time of diagnosis and with the inclusion of patients under clinical trial protocols. For further information about biospecimen collection, processing, quality control and biomarker assessment, please refer to [3] or to TCGA website (http://cancergenome.nih.gov). 2) To validate the HSP clusters detected in the TCGA dataset, the clinical information and the normalized gene expression levels of 1981 tumours from patients with breast cancer were acquired from the METABRIC cohort. [13]. The METABRIC database analyses 49,576 transcripts with Illumina HT 12 microarray technology and reports patient overall survival and disease-specific survival. These data were accessed through Synapse (synapse.sagebase.org, ID: syn1757063, syn1757053 and syn1757055).

The analysis workflow is summarized in Additional file 2. All analyses and graphs were performed using R software environment unless otherwise specified. This study has been approved by the Bioethical Committee of the Medical School of the National University of Cuyo, Mendoza, Argentina (0029963/2015).

### Intrinsic subtype classification

The expression levels of the PAM50 panel genes from each of the 1097 samples from TCGA were used to carry out the intrinsic subtype classification of tumours [2] which was performed using the “Bioclassifier” package, kindly given by Dr. K. Hoadley of the University of North Carolina Chapel Hill and available online. To perform this task, the normalized expression profile (normalized RNA_SeqV2 RSEM) of the 50 specific genes was used. Many of these genes are strongly related to BRCA behaviour and include ESR1, ERBB2, PGR, and MKI67 among others. To normalize the expression values from each gene the log_2_ expression levels were obtained and subsequently the median expression value of a subset of samples (50% oestrogen receptor positive and 50% oestrogen receptor negative population defined by immunohistochemistry) was subtracted. Once the samples were classified, principal component analysis, class to centroid correlation, and hierarchical cluster evaluations were performed to assess the quality and validity of the classification (Additional files 3 and 4). We found 89 and 100% concordances with previously reported classifications by Koboldt [3] and Ciriello [14] respectively (Additional file 1: Table S5). From the total samples analysed from the TCGA cohort, we found few cases of the Normal-like subtype (only 3.6%), 51.5% were Luminal A, 20% were Luminal B, 17% were Basal, and 7.5% were HER2, which are in agreement with other studies [15, 16]. All 1981 METABRIC patients were classified according to the PAM50 classification as described above and Normal-like patients were excluded from further consideration.

### Differential gene expression of TCGA samples

To evaluate differentially expressed genes (DEG) two different statistical packages, DESeq2 [17] and EdgeR [18], were chosen due to their demonstrated good performance [19]. In this study, we used raw count expression of 20,531 genes from 1211 tissue samples. We grouped samples according to the subtypes assigned, and then each group was compared against normal tissue expression profiles using the standard workflow as presented in: https://www.bioconductor.org/packages/3.3/bioc/vignettes/DESeq2/inst/doc/DESeq2.pdf and https://bioconductor.org/packages/release/bioc/vignettes/edgeR/inst/doc/edgeRUsersGuide.pdf. In both cases log_2_ fold change values were obtained associated with *P* values and False Discovery Rate values (FDR, a modified P value to correct the eventually false positives) by Benjamini and Hochberg method [20]. Results from DESeq2 and EdgeR are summarized in Additional file 5. The consistency between both methods was compared by Pearson’s correlation coefficient (mean correlation between methods 0.948 ± 0.01 SD) and Bland Altman analysis [21] (mean difference between methods of 0.02 and 97.37% of the measurements within the 95% confidence interval), which evidence high agreement between both techniques (Additional file 6). We detected a disagreement between both methods in at least one BRCA subtype in six genes (CRYAA, DNAJB13, DNAJC5G, HSPA6, HSPB3 and ODF1), all of which presented low expression levels. EdgeR runs with least computational resources than DESeq2, this motivated its preferential use. EdgeR ANOVA-like test was used to analyse differential gene expression within PAM50 subtypes and HSP-Clusters (Additional file 7).

### Heatmap construction and cluster analysis

The values of logarithm base 2 of normalized RSEM (RNAseq) plus 1 from 1033 patients (males, Normal-like tumours, and patients without clinical data were excluded) from the TCGA cohort were used to construct the HSPs expression matrix. The rows and columns were sorted based on a hierarchical cluster with average linkage and Pearson’s correlation distance. According to Silhouette dendrograms analysis (Additional file 8) patients were grouped into three clusters: HSP-Clust I, HSP-Clust II and HSP-Clust III.

### Survival model

The survivals analysis was performed according to REMARK guidelines [22]. The effect of each HSP on survival was estimated using a univariate Cox proportional hazard model with the survival information of the 1033 patients of the TCGA cohort considered in the heatmap graphic and cluster analysis. To correct for multiple testing FDR testing was conducted by Benjamini and Hochberg method. Once each patient of the TCGA and the METABRIC training and test set were classified into one of the three HSP clusters, Kaplan-Meier curves for each group were generated and the survival distribution was compared using Log-Rank test. A multivariate Cox proportional hazard model was used to determine statistically significant survival difference between clusters of TCGA cohort. The model was adjusted to several known prognostic predictors (inclusion criteria): lymph node status, tumour size, age, tumour stage, and PAM50 subtypes. As exclusion criteria we considered: males, patients with unknown metastatic status at the time of diagnosis, and Normal-like subtypes. From this filtering 1003 patients were left, with 81 events registered. The sample size was not considered a priori and all available patient data within inclusion criteria were considered.

### Nearest centroid classifier

To train a HSP single-sample-predictor with the METABRIC dataset**,** samples gene expression levels were scaled and only probes that were associated with the 95 HSPs where used in the classifier. In cases where there was more than one probe matching a single gene, all probes values were averaged and collapse into one. From the 95 HSP genes, HSPA7 did not match to any of the probes analysed and those HSP genes that presented low expression levels in the TCGA cohort (DNAJB8, DNAJC5G, DNAJB3, ODF1, CRYAA and HSPB3) were not considered to train the classifier. The dataset was randomly divided into a training set (*n* = 915) and a test set (*n* = 914), then a hierarchical clustering algorithm with average linkage and Pearson’s correlation distance was applied to the training dataset and the resulting dendrogram tree was cut to divide the set of patients into three different HSPs expression profile groups. From each cluster, the corresponding centroid vector was calculated and the samples in the test set were labelled according to the class centroid from which each sample presented highest Spearman correlation.

## Results

### Transcriptomic analysis evaluating the RNA expression profile in TCGA BRCA cohort

**Fig. 1:**
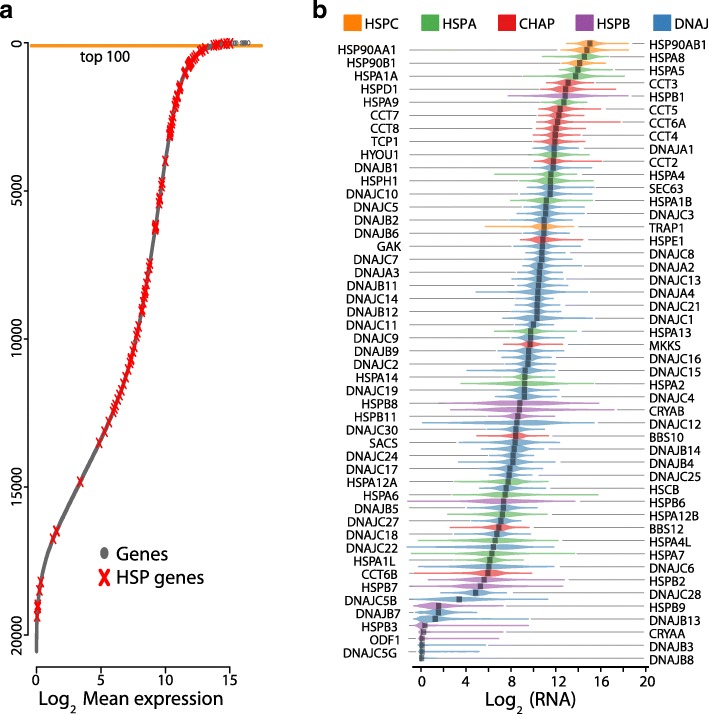
HSPs expression in breast cancer. **a**) The mean expression of each gene in all cancer samples was calculated and sorted in decreasing order. HSP genes were localized with a red x. Note that six HSP genes are above the orange line of the top 100 expressed genes. **b**) The graphs show the RNA expression distribution of HSP genes in the cohort. Note that figure is thicker were the values are more frequent

We first evaluated the absolute normalized expression levels of the 95 HSP genes. The overall trend indicates that HSPs were highly expressed in tumour samples (one-sided Mann-Whitney U test P val = 1.256e^− 10^), nevertheless, a more detailed study showed a group of six genes (DNAJB8, DNAJC5G, DNAJB3, ODF1, CRYAA, and HSPB3) with very low expression levels in almost all the samples and was not detected in at least 50% of the cohort or more. On the other hand, six HSPs (HSP90AB1, HSP90AA1, HSPA8, HSP90B1, HSPA5, and HSPA1A) were ranked in the top 100 most expressed mRNAs of BRCA (hypergeometric test P val = 4.09^− 07^). (Fig. 1 and Additional file 1: Table S2). The rest of the HSPs were distributed in a wide range of expression. Almost all members of the Chaperonin subfamily (TCP1, CCT2, CCT3, CCT4, CCT5, CCT6A, CCT7, and CCT8) were also expressed at similarly high levels. It is important to note that the HSPB subfamily, except HSPB1, appeared with low transcript expression levels.

**Fig. 2:**
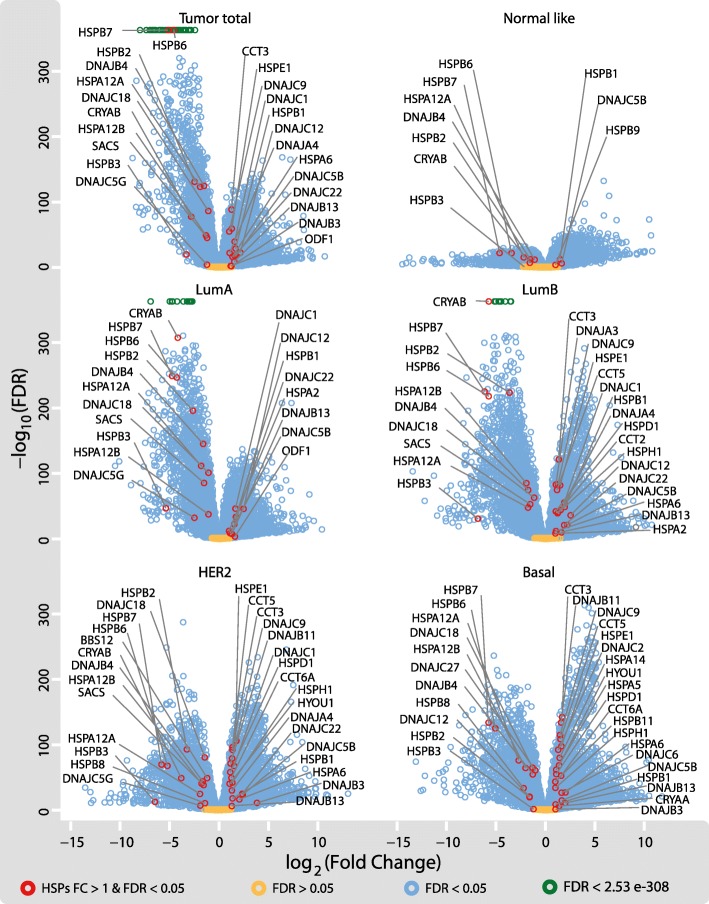
Differential expression of total genes in breast cancer. Volcano plots of genes expression analysis accomplished by Edge R method. In the x-axis the log_2_ fold change respect to normal tissue is represented, while in y-axis the -log_10_ of FDR is shown (the higher values show smaller FDR). Observe that HSP genes with log_2_ fold change > 1 and FDR < 0.05 are indicated as red circles. The green symbols at the top of the subpanels indicate genes with very small FDR (FDR < 5e^− 324^). Significant fold changes of non-HSP genes are light blue coloured

We continued the analysis evaluating DEG comparing BRCA tissues against normal tissues. In this study we considered only genes that showed absolute values of log_2_ fold change (log_2_FC) > 1 and statistical significance (FDR < 0.05). The tabulated results (Additional file 5) show that in BRCA there were 3994 upregulated and 2155 downregulated genes. To our knowledge, this is the first report of the DEG between tumours and normal tissues taking into account PAM50 groups of RNAseq BRCA data (1097 patients). With respect to HSP genes, 13 were upregulated and 11 were downregulated (Additional file 1: Table S3). Deregulation of HSP genes increased in BRCA subtypes as follows: Luminal A, Luminal B, HER2 and Basal. To achieve a better statistical interpretation volcano plots were used (Fig. 2). These graphs allow the contextualization of the HSP genes respect to the rest of the genes letting a complete appreciation of gene expression changes that were modulated differentially in the entire cohort (Fig. 2 tumour total) and between the intrinsic BRCA subtypes (Fig. 2). The patients were subdivided according to the PAM50 classification to investigate whether the intrinsic subtypes of BRCA manifested different expression of HSP genes. The PAM50 classification is a “single sample predictor” and classifies each of the samples in 5 tumour intrinsic subtypes [2]. From a total of 1097 samples 566 were classified as Luminal A, 217 as Luminal B, 82 as HER2-enriched, 192 corresponded to Basal and 40 were Normal-like (Additional file 1: Table S4). The comparison of the correlative immunohistochemical characteristics of each tumour was included; these results appeared congruent with the molecular classification (Additional file 1: Table S4). In the case of upregulated HSP genes, the log_2_ fold-change mean and standard deviations (SD) in the different subtypes ranged between 1.38 and 1.64 and 0.31 to 0.69 respectively; the downregulated genes showed log_2_ fold-change mean in the range of 2.34 to 3.62 and were more dispersed (SD = 1.36 to 2.26) compared to upregulated genes. Surprisingly we found that several HSPs were within the first hundred genes with the lowest FDR values in Luminal A and Luminal B, which points out that some HSPs DEG in BRCA shows remarkable steady differences between normal and tumour samples.

**Fig. 3:**
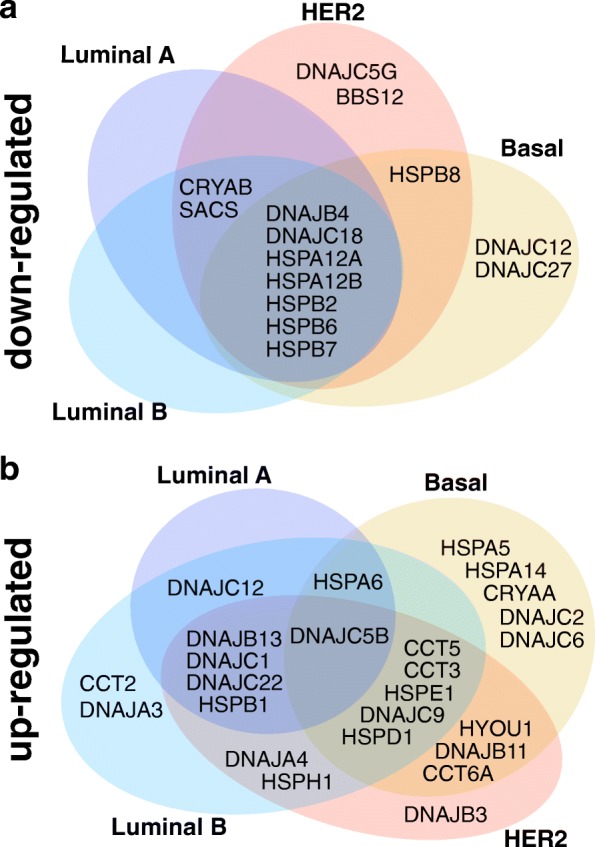
Venn diagrams showing overlapped and specific differentially expressed HSPs in intrinsic subtypes of breast cancer. The figure shows a summary of HSP genes expression analysis performed by Edge R method (fold-change > 2, FDR-adjusted *P* values < 0.05, and with no disagreement mean between the EdgeR and DESeq2 methods). Normal group was discarded based on the low number of cases. **a** Down-regulated HSP genes. **b** Up-regulated HSP genes

After exploring HSPs expression changes, we found many deregulated HSP genes, some of which were specific for certain molecular subtypes while others were shared by different intrinsic subtypes (Fig. 3). In particular, this analysis revealed that 38 of the 95 HSP genes were found differentially expressed. In the case of downregulated genes, a group (DNAJB4, DNAJC18, HSPA12A, HSPA12B, HSPB2, HSPB6 and HSPB7) presented decreased transcript levels in all BRCA molecular subtypes while some HSPs showed subtype specific downregulation (DNAJC27 and DNAJC12 in Basal and BBS12 and DNAJC5G in HER2). Others HSPs presented decreased levels of transcripts shared between different subtypes (HSPB8 between HER2 and Basal and CRYAB and SACS between HER2, Luminal A and Luminal B tumours). Evaluating the upregulated genes, we found a more complex combination where only DNAJC5B was upregulated in all subtypes. HSPB1, DNAJB13, DNAJC1 and DNAJC22 were upregulated in all except in the Basal subtype. The Basal subtype showed the highest number of specific upregulated genes (DNAJC2, DNAJC6, HSPA5, HSPA14 and CRYAA), DNAJA3 and CCT2 were upregulated in Luminal B, and DNAJB3 was only upregulated in HER2 tumours. Luminal A did not have any specific upregulated HSP.

### Fold change expression values of the different HSP subfamily

**Fig. 4:**
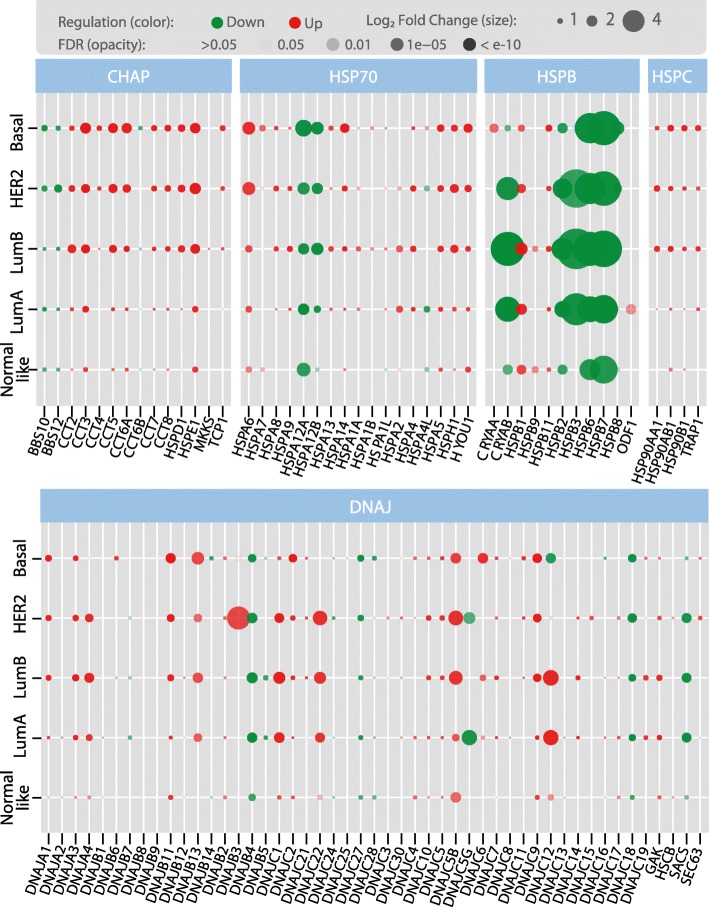
Diagram showing a summary of HSPs expression grouped in subfamilies in breast cancer according to the intrinsic molecular subtypes. In the figure, the diameter of the circles shows the log_2_ fold change assessed by EdgeR method. The circles in green show downregulated genes and the red ones represent upregulated genes. The circle opacity is related to the FDR values, circles with FDR > 0.05 are transparent and therefore not depicted. The figure makes emphasis on fold change expression values regardless any threshold

We next proceeded to compare the magnitude the HSPs DEG pattern in the BRCA tissues arranging the HSPs in their five subfamilies. Figure 4 shows that the CHAP subfamily (14 members) appeared upregulated in BRCA with only three members (BBS10, BBS12 and in a lesser degree CCT6B) downregulated. In this figure we can also see that most of the HSP70 subfamily members were upregulated while only two members (HSPA12A and HSPA12B) were strongly downregulated. HSPA4L showed a particular profile, its expression decreased in HER2 and Luminal A cancers only. The study of the HSPB subfamily showed interesting characteristics. Incremented transcripts levels of HSPB1, HSPB9 and HSPB11 were observed in most BRCA subtypes, CRYAA was upregulated only in Basal subtype and ODF1 showed an increased expression in Luminal A tumours that was not significant by the Deseq2 method. Interestingly, the genes CRYAB, HSPB2, HSPB6 and HSPB7 were strongly downregulated in all BRCA subtypes. The HSPC subfamily involves HSP90 genes with well-known clinical implications in cancer [23]. HSPC members showed mild positive fold changes in all BRCA subtypes. It is of interest to mention that several HSP genes have relatively high expression levels in normal tissues, therefore in these cases fold changes in expression levels between normal and cancer tissues are less pronounced but could be of important biological significance. (e.g. HSP90AA1 have a fold change of 0.98). The large DNAJ subfamily revealed a mixed behaviour, some members (DNAJA2, DNAJB1, DNAJB8, DNAJB9, DNAJC8, DNAJC25) showed null variations, others were upregulated (DNAJA1, DNAJA3, DNAJA4, DNAJB2, DNAJB11, DNAJC1, DNAJC2, DNAJC5, DNAJC5B, DNAJC9, DNAJC10 and GAK) and some were downregulated (DNAJB4, DNAJC18, DNAJC27, DNAJC28 and SACS) in all subtypes. Several interesting expression profiles of DNAJ members need to be especially mentioned. For example, DNAJC12 appeared strongly upregulated in Luminal A and B, in contrast to the Basal subtype where this gene appeared downregulated. DNAJB3 transcripts appeared strongly upregulated in the HER2 BRCA subtype and DNAJC22 appeared upregulated in Luminal A, Luminal B and HER2 subtypes. A summary of the HSP subfamilies fold change trends across PAM50 classes is depicted in Additional file 9 to grasp a better understanding of the HSP groups changes and variability in the different subtypes which reveals that a complex regulation is active on every HSP subfamily, even for members of the same group.

Beyond particular cases, less marked but important differences were found in the overall expression patterns of HSP gene families between subtypes. Primarily, HSPH (from the HSP70 superfamily), HSP90 (HSPC), and type I and type II chaperonins (from the CHAP family) were found expressed at higher levels in Luminal B, HER2 and Basal tumours than in Luminal A subtypes, while for the HSPB family, Basal tumours showed an overall less marked decrease of these group of genes with respect to normal tissue, which represents greater expression of them with respect to the rest of the subtypes, especially in relation to HER2 and Luminal B types (Additional file 10 A).

### HSPs expression variability and clinical outcome

To investigate whether the complex regulation of HSP genes was associated with clinical outcome, we performed an integrated transcriptomic analysis of the 95 HSP genes in the TCGA BRCA patients with known follow-up (*n* = 1033; Normal-like subtypes excluded). It is well-known that several HSPs have clinical correlates, the best example is probably HSP90AA1 that it is used as an adverse prognostic factor not only in BRCA but also in other cancers [23]. In order to get further information of the clinical relevance of HSPs, we performed an overall survival analysis by Cox univariate model based on the expression levels of each HSP. We observed 23 HSP genes with clinical statistical significance from which five genes were associated with a good prognosis (HSPA2, DNAJB5, HSCB, HSPA12B and DNAJC4) and 18 (CCT6A, DNAJA2, HSPA14, CCT7, HSPD1, CCT2, HSPA4, DNAJC6, CCT5, SEC63, HSPH1, CCT8, CCT4, HSP90AA1, HSPA8, DNAJC13, HSPA9 and TCP1) with a poor prognosis (Table 1).

**Table 1:** Univariate Cox proportional hazard risk of breast cancer based on HSP expression. Regression coefficients, hazard risk coefficients, standard error, *P* value and FDR are presented. Only HSP genes with FDR < 0.05 are shown

Gene	Coefficient	HR	Coeff SE	*P*-val	FDR
HSPA2	−0.35	0.71	0.10	< 0.001	0.005
DNAJB5	−0.32	0.73	0.10	0.002	0.011
HSCB	−0.29	0.75	0.11	0.009	0.037
HSPA12B	−0.29	0.75	0.10	0.003	0.016
DNAJC4	−0.27	0.76	0.10	0.006	0.027
CCT6A	0.22	1.25	0.08	0.009	0.037
DNAJA2	0.25	1.29	0.08	0.002	0.011
HSPA14	0.27	1.32	0.09	0.001	0.009
CCT7	0.28	1.32	0.11	0.008	0.034
HSPD1	0.30	1.35	0.10	0.003	0.013
CCT2	0.30	1.35	0.08	< 0.001	0.001
HSPA4	0.31	1.36	0.11	0.005	0.025
DNAJC6	0.34	1.40	0.11	0.002	0.011
CCT5	0.35	1.42	0.10	< 0.001	0.005
SEC63	0.35	1.42	0.09	< 0.001	< 0.001
HSPH1	0.35	1.42	0.10	< 0.001	0.004
CCT8	0.40	1.49	0.10	< 0.001	0.001
CCT4	0.40	1.49	0.10	< 0.001	< 0.001
HSP90AA1	0.40	1.49	0.09	< 0.001	< 0.001
HSPA8	0.41	1.51	0.12	< 0.001	0.004
DNAJC13	0.46	1.58	0.11	< 0.001	< 0.001
HSPA9	0.46	1.58	0.10	< 0.001	< 0.001
TCP1	0.50	1.64	0.10	< 0.001	< 0.001

**Fig. 5:**
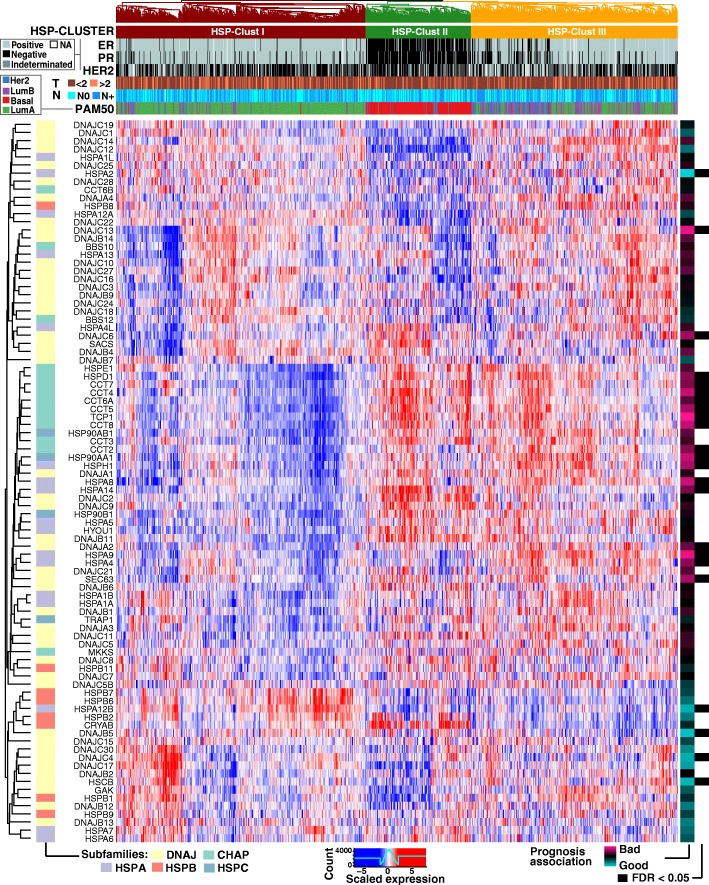
HSPs gene expression heatmap of TCGA BRCA cohorts. Expression patterns of 89 HSP genes in 1033 samples are depicted (central panel, low expression levels in blue and high expression levels in red). By a hierarchical clustering algorithm patients were group into HSP-Clust I (red), HSP-Clust II (green) and HSP-Clust III (orange) (upper dendrogram). Several rows were added to indicate: immunohistochemical status of receptors (ER, PR and HER2), tumour size (*T* > 2 cm or *T* < 2 cm), satellite nodules spread (N positive or N negative) and PAM50 classification. We also added three columns indicating HSP corresponding subfamilies, univariate Cox’s regression model coefficients (pink represents positives coefficients (bad prognosis), while light blue are negatives coefficients (good prognosis)) and its corresponding FDR values (black boxes represent FDR value for Cox’s coefficients < 0.05)

**Fig. 6:**
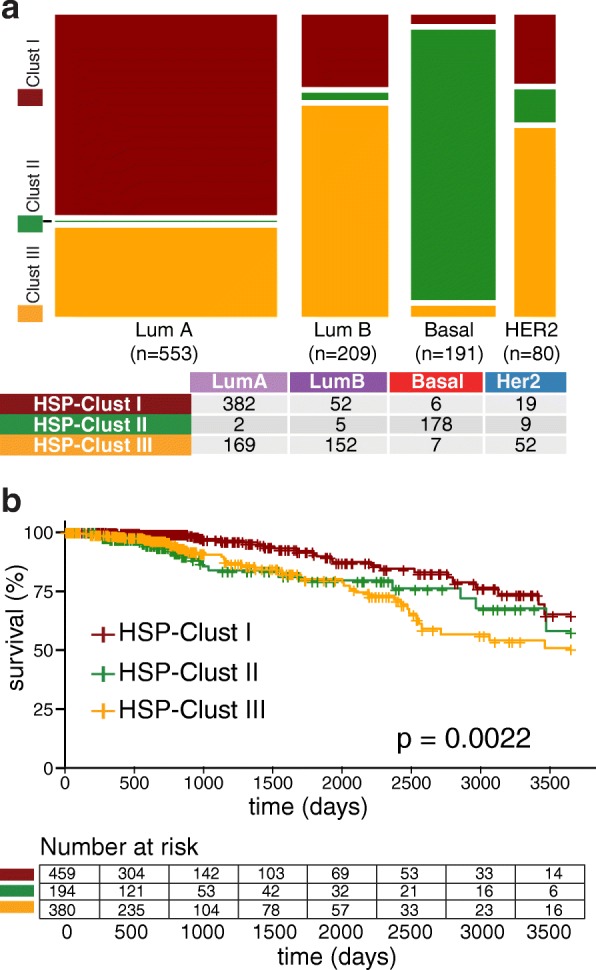
HSP cluster characterization. **a**) Agreement between PAM50 and HSP clusters. The size of the bars is in proportion to the number of samples in each category. **b**) Overall survival of HSP clusters. Kaplan-Meier curves corresponding to HSP-Clust I, HSP-Clust II and HSP-Clust III. Statistical significance was evaluated by Log-Rank test

**Fig. 7:**
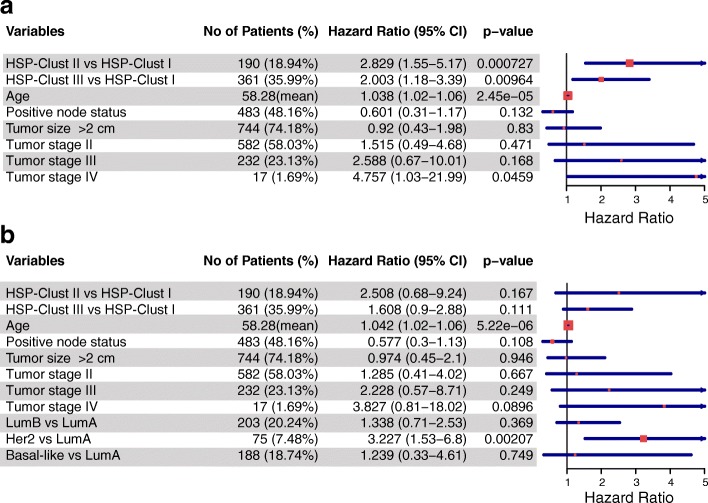
Multivariable Cox Model of HSP-Clusts. **a**) Forest plot showing the hazard risk of HSP-Clusters controlling for confounders (age, node status, tumour size, tumour stage). Hazard ratios, 95% confidence interval and corresponding *P* values are depicted. **b**) Same Cox’s model plus de addition of PAM50 subtypes as covariates

Next, we explored whether the BRCA patients could be grouped into clinically relevant clusters based on HSPs expression patterns. To test this hypothesis we performed an unsupervised hierarchical cluster analysis that separated the TCGA cohort into three main branches (Fig. 5). The three groups were called HSP-Clust I (red in Fig. 5), HSP-Clust II (green) and HSP-Clust III (orange). These three HSP clusters corresponded to PAM50 classification as follows: the HSP-Clust I had 83% of Luminal A tumours, HSP-Clust II was composed mainly by Basal-like tumours (92%), and the HSP-Clust III was the most heterogeneous group with 44% of Luminal A tumours and 40% of Luminal B tumours (Fig. 6a). The HER2 subtype was dispersed into the three HSP groups, but the majority were seen in the HSP-Clust III. The Kaplan-Meier curves of the HSP clusters showed highly significant differences in overall survival between groups (Fig. 6b, *P* = 0.0022), letting us identify a low-risk group (HSP-Clust I) and a high-risk group (HSP-Clust III). Multivariable analyses of HSP-Clust I against HSP-Clust II and HSP-Clust III adjusted for known clinical covariates (tumour size, node status, age, and tumour stage) showed different survival rates for the HSP-Clust II, with a hazard ratio = 2.829 (CI 95% = 1.55–5.17) and *P* value = 0.0007; and HSP-Clust III hazard ratio = 2.003 (CI 95% = 1.18–3.39) and P value = 0.01 (Fig. 7a). We also tested a model including the intrinsic molecular subtypes.In this case the *P* values of HSP-Clust coefficients became non-significant (Fig. 7b), which suggests that HSP-Clusts effect on survival is related to PAM50 subtypes. In order to validate the HSP-Clusts found, we used the METABRIC cohort divided in a training and test set to reproduce our results. Briefly, by a hierarchical cluster algorithm we divided the training set into three distinct groups which were consistent with the HSP-Clusts found in the TCGA dataset (Additional file 11 A) (TCGA HSP-Clust I vs. METABRIC HSP-Clust I with a correlation factor = 0.87, TCGA HSP-Clust II vs. METABRIC HSP-Clust II with a correlation factor = 0.82 and TCGA HSP-Clust III vs. METABRIC HSP-Clust III with a correlation factor = 0.7). Centroids for each HSP-Clusts from the training set were used to classify samples from the test set. The centroids obtained from the test sets were in agreement with the others centroids (Additional file 11 A). The PAM50 subtype distribution regarding HSP-Clusts was similar in both sets (Additional file 11 B). The overall survival of the HSP-Clusts corresponding to training and test sets showed a significant difference between HSP groups (both training and test set had a Log-Rank test with a *P* value < 0.0001) (Additional file 11 C).

It is interesting to note that there is a significant (but not complete) overlap between BRCA PAM50 intrinsic subtypes and HSP-Clusts. For instance, HSP-Clust I is enriched with Luminal A tumours and also presents lower expression levels of HSPH, HSPC and type I and II chaperonins compared to HSP-Clust II and HSP-Clust III, which are enriched with Basal and Luminal B tumours respectively. HSP-Clust II presents significantly higher levels of some HSPB genes such as HSPB2, HSPB3, CRYAA and CRYAB compared to the others HSP subtypes (a pattern that was also observed in Basal-like tumours). HSP-Clust III is enriched with DNAJA gene expression (similar to the Luminal B and HER2 subtypes) (Additional file 10 B).

## Discussion

This is the first comprehensive study examining the whole HSP family in breast cancer patients. The HSP family, characterized by 95 genes and one pseudogene, represents only 0.46% of the 20,531 analysed genes. In this study, we found that in BRCA almost 30% of the total genes were deregulated (19.45% upregulated and 10.5% downregulated), where the HSP family accounts for 0.39% of this deregulation (0.32% of the upregulated genes and 0.52% of the downregulated). Several reasons have been mentioned to explain HSP misregulation in cancer: by the stressful situations found in cancer tissues [4], to increase the stabilization of transcription factors, receptors, protein kinases and other proteins that lie along the pathways of normal to cancer transition [24], and by the oncogenic agents/events that directly affect the heat shock response [25]. The activation of Heat Shock factors (HSF) during cancer progression can in turn explain the activation of the HSPs molecular chaperones [26, 27]. Therefore, considering that cancer tissues are subjected to several stressful situations we expected to see more upregulated HSPs (*n* = 13) and fewer downregulated (*n* = 11). At this point we have to say that the expression levels of several HSPs were very close to the cut-point used (log_2_ fold-change = ±1), this happened for example with the HSPC family which codes for the HSP90 (all appeared with a certain level of upregulation, see Fig. 3). In any case, it is evident that in BRCA the expression levels of several HSP family members are affected. Upregulation was noted mainly in the CHAP and HSPC family members while the greatest downregulation was observed in most HSPB members (Fig. 3 and Additional file 9). The downregulation of the small HSPs agrees with a recent report [28]. The HSP70 superfamily (which includes the HSP70 and HSP110 or HSPH family) and the DNAJ members showed variable results with ups and downs.

The present study revealed that deregulation of the HSPs varied according to the BRCA molecular subtype. Of importance at this point is: what are the functional implications of the up- and down-regulation of the HSP genes in each breast cancer subtypes? This is not an easy point to address because in the present report we are finding alterations in HSP genes that are little known to be linked with breast cancer; moreover others like DNAJB3 (increased in HER2 subtype), DNAJB13 and DNAJC22 (increased in Luminal and Basal subtypes), and SACS (increased in all subtypes) have not been related with any cancer type. Let’s begin with the Chaperonin family. The members of this group can be divided into three distinct subgroups: Type I chaperonins, established by HSPE1 and HSPD1 genes (also known by their bacterial names GroES and GroEL or HSP10 and HSP60 respectively), type II chaperonins forming the T-complex protein-1 ring complex (TRiC) which is formed by a double ring structure with eight distinct subunits (TCP1 and CCT genes) working as an ATP dependent protein folding machinery [29], and finally the BBS group of genes (BBS10, BBS12 and MKKS) that in conjunction with the TRiC complex mediate the BBSome assembly [30]. Of this group of genes, HSPD1, HSPE1, CCT3 and CCT5 were overexpressed in Basal, HER2 and Luminal B subtypes (more aggressive BRCA tumours). HSPD1 and HSPE1 are located on chromosome 2 arranged in a head-to-head orientation and both are implicated in macromolecular protein assembly and mitochondrial protein import, while CCT3 and CCT5 form a protein complex folding various proteins including actin and tubulin upon ATP hydrolysis and, as part of the BBS/CCT complex, they are involved in the assembly of the BBSome, which in turn is implicated in ciliogenesis regulating transports vesicles to the cilia [30]. At this point we have to remember that breast cancer cells, mainly stem cells, have primary cilia (a non-motile microtubule based cell-surface organelle) that acts as a cellular antenna for receiving signaling pathways involved in the regulation of cell proliferation, differentiation and migration [31, 32]. Therefore our study adds evidence to an important role of CCT3 and CCT5 in the more aggressive BRCA tumours: Basal, HER2 and Luminal B subtypes. CCT3 has been involved in mitosis progression and associated with poor prognosis in hepatocellular carcinoma [33], has been implicated in osteosarcoma tumorigenesis [34], and appeared as a candidate biomarker in epithelial ovarian cancer [35] and in cholangiocarcinoma patients [36]. CCT3 was found differentially expressed in colon and other epithelial cancers [37] and its expression has been associated with drug resistance in a squamous lung cancer cell line [38]. CCT5 was found upregulated in p53-mutated breast tumours and might be implicated in resistance to docetaxel treatment [39]. Of notice, all the other TRiC genes except CCT6B were also among the most highly expressed in cancer and upregulated accordingly in the different subtypes, suggesting an important role of the TRiC complex specifically in BRCA as previously suggested [40]. TRiC has an essential role in cell proteostasis in physiological conditions but also in oncogenesis and cancer progression [41] and is known to regulate the proper folding of several others genes involved in cancer such as actin, tubulin [42], p53 [43] and protoncogene STAT3 [44]. In our study, HSP-Clust II (enriched with Basal-like tumours) presented high expression levels of the TRiC complex genes. The current standard of treatment of triple-negative (TNBC) tumours is systemic neoadjuvant chemotherapy that typically include taxanes which inhibit tubulin depolymerization [45]. We hypothesize that the measurement of the TRiC complex genes along with the classification of tumour samples in the different HSP-Clusts could be used as an important tool to predict taxane response, even though further studies are needed to validate this assumption.

Coming back to HSPE1, in a previous proteomic analysis this protein appeared with altered expression in MDA-MB-231 breast cancer cells (triple negative highly aggressive cells) [46] and both HSPD1/HSPE1 have also been found upregulated in other cancer types associated with tumour cell transformation [47]. Interestingly, both TRiC genes and HSPD1/HSPE1 were co-expressed and were associated with worst prognosis individually and had high expression in the HSP-Clust II and III of our study (Additional file 10 B). All this data together suggest that not only the TRiC complex has a protagonist role in cancer behaviour but also that the HSPD1/HSPE1 complex is involved tightly with TRiC in proteostasis regulation, an association that is poorly understood in breast cancer and should be further studied. On the other hand, BBS12 was underexpressed in the HER2 subtype predominantly and along with BBS10, both showed decreased expression levels in all subtypes. MKKS gene (also known as BBS6) was not altered. Therefore, our study reveals specific chaperones that participate in the assembly of the BBSome altered in BRCA.

The HSP70 family is a group of evolutionary conserved and ubiquitously expressed genes that in conjunction with the DNAJ family act as a protein folding regulatory network that also protects the cell against stressful conditions [48]. Several members of the HSP70 family were found highly expressed (HSPA8, HSPA5, HSPA1A) or upregulated in BRCA. We found that HSPA6 expression appeared elevated mainly in Luminal A, Luminal B and Basal subtypes. In a previous study high levels of this protein were associated with recurrence in hepatocellular carcinoma [49]. HYOU1 also known as oxygen-regulated protein 150 (ORP150) was upregulated in HER2 and Basal subtypes and the protein has been implicated with tumour progression in different cancers [50–53]. HSPA5 was found highly expressed in all subtypes, and especially upregulated in Basal tumours in our study, and has been associated with endoplasmic reticulum stress response (ERSR), inhibition of apoptosis and autophagy in several studies [54–56]. HSPA8 was the most expressed gene of the HSP70 family and one of the genes with the strongest association with survival in our study. This gene is constitutively expressed and has been largely associated with the protein folding and stress response [57, 58]. Interestingly, DNAJC12, a gene strongly upregulated in Luminal A and B tumours, was found to interact with HSPA8 under ERSR [59].

Only one HSP appeared upregulated in the four subtypes considered: the protein encoded by DNAJC5B, which is implicated in protein processing at the level of the endoplasmic reticulum [60]. This protein has been found in secretory vesicles as well as in synaptic and clathrin-coated vesicles in neuroendocrine, exocrine and nervous cells. Of interest is that this member of the DNAJ family has been found upregulated in human bladder carcinoma, gastric adenocarcinoma, and glioblastoma cell lines by the OCT4B1 variant (octamer-binding transcription factor 4 B1 variant) which is expressed by pluripotent normal and cancer stem cell lines and linked to anti-apoptosis [61]. In addition, these authors found that the OCT4B1 variant is also linked to upregulation of the chaperonin DNAJC11 which is complexed with mitofilin in the mitochondrial membrane [62] and has been associated with neuromuscular diseases and lymphoid abnormalities [63]. In this study, DNAJC11 appeared slightly upregulated in Luminal B, HER2 and Basal subtypes. No attention has been directed to these proteins (DNAJC5B and DNAJC11) in BRCA. It is now evident that further studies must be directed to clarify the role of these proteins. DNAJC9 appeared upregulated in Basal, HER2 and Luminal B, and in previous studies has been found upregulated in node-positive uterine cervical carcinoma [64].

Our study revealed HSPs that appeared both deregulated and not well studied in BRCA; for example, DNAJB3 appeared with high levels of upregulation only in HER2 BRCA subtype. Close gene location with HER2 gene cannot explain upregulation of DNAJB3 since this gene is located on chromosome 2 while HER2 (amplified in HER2 subtype) is located on chromosome 17. Little is known about the protein encoded by this gene, and its role in cancer in general and in breast cancer in particular is not known. DNAJB3 has been reported downregulated in obese human subjects, DNAJB3 over-expression in adipose cell lines caused: a) reduction in JNK (Jun N-terminal kinase) improving insulin sensitivity and enhancing glucose uptake and b) mediated PI3K/AKT pathway activation [65]. Of interest here is that the PI3K/Akt signalling pathway is negatively regulated by PTEN and we have reported that PTEN is downregulated by HSPB1 (HSP27), both proteins have been implicated in HER2-positive tumours [66]. Therefore, it will be of interest to study the role of DNAJB3 in HER2 BRCA. However, we have to take into account that the upregulation levels of this gene might appear statistically significant, but the number of RNA molecules could be relatively low. Therefore, an upregulated gene could have few RNA copy numbers and we ignore if the encoded protein has biological significance. Nevertheless, this entire complex HSP70/DNAJ landscape suggests an intricate regulatory interaction between these genes that remains to be untangled.

Finally, among the upregulated small heat shock proteins, HSPB1 stands out as the highest expressed of the group and appeared upregulated in Luminal A, Luminal B, and HER2 (close to the cut-point in Basal); the protein encoded by this gene has been well studied in breast cancer [4, 67].

Many of these upregulated genes and proteins have been reported as associated with tumour progression in different cancer types and in several opportunities with poor prognosis. In concordance, we have found that some of these genes appeared upregulated mainly in aggressive breast cancer subtypes that were clustered in the HSP-Clust III group. Moreover, the complexity of the regulation of the HSPs in BRCA is further increased when we consider the high number of client proteins that are associated with the HSPs [11].

Another interesting observation from the present study is that several HSPs were downregulated in all breast cancer subtypes: DNAJB4, DNAJC18, HSPA12A, HSPA12B, HSPB2, HSPB6, and HSPB7. DNAJB4 is a member of the DNAJ family and is described as a tumour suppressor [68], which is in agreement with our results; increased expression of DNAJB4 has been implicated in the stabilization of wild-type E-cadherin (but not the mutant) stimulating the anti-invasive function of E-cadherin in gastric cancer cells [68]. Little is known about the protein coded by DNAJC18, but a polymorphic variant has been associated with aggressive bladder carcinoma [69]. HSPA12A encodes a protein of the HSP70 family that seems to act like a protective factor in gastric cancer [70]. We found high levels of suppression in several members of the HSPB family (CRYAB, HSPB2, HSPB6 and HSPB7) (Fig. 3); in an integrated genomic and epigenomic analysis the ATM, HSPB2 and CRYAB (this last downregulated in Luminal A, Luminal B and Basal) genes were found commonly deleted and underexpressed in patients with breast cancer brain metastasis [71]. The role of CRYAB gene (Alpha B-crystallin HSPB5) is controversial in cancer [72–79], its expression has been associated with aggressive breast cancer subtypes. In agreement with our results, HSPB6 and HSPB7 have been found downregulated in several tumour types [80–85], and we report here this downregulation in all subtypes of BRCA is possibly supporting a role as tumour suppressor genes. In our analyses we compared tumour tissue with normal breast tissue, but displacement of stroma in the tumour samples could be affecting the results. Nevertheless, in a recent publication none of the HSP genes were found altered by the confounding effect of tumour purity [86]. The HSPs expression patterns of the molecular subtypes are still heterogeneous [15] and the results of the present study contribute to the characterization of these subtypes. We are now completing the study of the methylation status of the HSP genes as well as the mutations, amplifications and deletions in these genes.

Of importance, we have to mention that some genes evaluated in this work presented clinically and biologically meaningful characteristics already described, but some others genes are totally unknown at the moment [87]. The clinically important genes DNAJB5, HSCB, HSPA2 (usually differentially overexpressed in Luminal A and B), DNAJC4, and HSPA12B (downregulated in BRCA) presented a significant FDR value in the Cox’s proportional hazard model presenting negative coefficients (their expression was associated with a good prognosis). In contrast, the genes with high expression levels significantly associated with poor prognosis were: CCT6A, HSPA14, DNAJC6 (upregulated in Basal), CCT2 (upregulated in Luminal B), CCT5, HSPD1 (upregulated in Basal, Luminal B and HER2), SEC63 (upregulated in HER2), TCP1, CCT4, CCT7, CCT8 (upregulated in HER2 and Basal), HSP90AA1 (upregulated with a near 0.9 log_2_ fold-change in Luminal B, HER2 and Basal), HSPH1 (upregulated in Luminal B, HER2), DNAJA2, HSPA9, HSPA4, DNAJC13, and HSPA8. Many of which were previously mentioned (HSP90AA1, TRiC, HSPD1/HSPE1, HSP70 family) and others for which their role in BRCA has not been exhaustively studied.

An important point of this study is the finding of three discrete HSPs expression profiles with prognostic significance (*P* = 0.0022) that we called HSP-Clust I, II and III. These HSP clusters groups were reproduced in an independent dataset using the METABRIC cohort and a single sample predictor was trained to classify unknown samples into one of the three HSP-Clusts with robust results. Importantly, TCGA and METABRIC datasets were developed using different RNA measurement technologies but the clusters found showed striking similarities and had significant impact on disease outcome. An interesting point to address is that the HSP-Clust II (predominantly basal-like) in METABRIC is much more clearly associated with a poor prognosis than the same signatures in the TCGA, a plausible explanation might be found in the survival differences of Basal-like tumours in each cohort (Additional file 12). Even though HSP-Clusts survival is highly related to PAM50 subtypes as expected, it is important to notice that the overlap between groups is not complete. Regarding Luminal tumours, HSP-Clust I presented mainly Luminal A tumours while HSP-Clust III presented mixed proportions of Luminal A and Luminal B subtypes. These findings could be reflecting differences in the biology of Luminal A tumours from HSP-Clust I with respect to Luminal A tumours of HSP-Clust III. Also, since HSPs have been long related with drug resistance, it would be of interest to test if the different HSP-Clust are related with different chemotherapy response profiles, which in turn, could imply a differential treatment for each HSP-Clust group. Further studies will be necessary to turn this classification useful for clinical practice and to better characterize the prognostic and treatment for these groups of patients. Since we used a combination of all HSP genes to evaluate survival, this could add superfluous information that can reduce the performance of the study. It will be interesting to reduce the number of HSP genes in order to increase the potential of the HSPs expression patterns as a prognostic factor. For instance, the clinical subset of HSP genes with clinical importance could be used as a genetic signature to develop prognostic tests or as a base for future research of predictive assays based on immunohistochemistry, microarray or rPCR.

## Conclusions

Our results show the existence of several HSP genes deregulated in all molecular subtypes of breast cancer while others appeared deregulated in specific molecular subtypes. We also found that the overall survival of breast cancer patients appeared associated with the expression level of certain HSPs.

## Additional files

Additional file 1: **Table S1.** Clinical data of TCGA patients. The data was updated with the available follow up information (May, 2015). **Table S2.** Gene mean expression in breast cancer tissues. The mean expression of each gene in all cancer samples was calculated and sorted in decreasing order. **Table S3.** Summary of misregulated genes in BRCA. Tabulated data show the number and percentages of total genes and HSP genes presenting > 2 fold-change in total samples and according to intrinsic BRCA subtypes. **Table S4.** Summary of PAM50 classification and immunohistochemical characteristics of tumours. (XLSX 1080 kb)

Additional file 2: Data analysis workflow. Schematic representation of HSPs transcriptomic and survival analysis process. (PDF 108 kb)

Additional file 3: PAM50 classification quality control of TCGA’s samples I. A) Principal components analysis of the training and test sets. Note the subtype clustering and the superposition between both datasets. B) Correlations between subtype assigned and the corresponding subtype centroids per sample and relation between subtypes and proliferation index. Each dot represents a single sample. (PDF 166 kb)

Additional file 4: PAM50 classification quality control of TCGA’s samples II. Unsupervised hierarchical clustering of samples according to PAM50 gene set expression. Note the consistency between the subtype assigned to each sample by PAM50 algorithm and the group composition determined by the clustering technique. (PDF 133 kb)

Additional file 5: Differential gene expression of 20,531 genes comparing cancer tissue against normal breast tissue. The values were determined by EdgeR and DESeq2 methods; also the analysis was performed according molecular subtype classification. For better data exploration HSP genes were separated in auxiliary tables. (XLSX 22435 kb)

Additional file 6: Fold-change consistency between EdgeR and DESeq2 methods. A) Correlation analysis between fold-change obtained by both methods. The figure shows a tight linear trend between EdgeR and DESeq2 fold-change estimations. Genes found significant for both methods are represented in yellow circles, in green and red are genes significantly differentially expressed by one of the two methods and in white, genes with no significant changes by both techniques. B) Bland Altman analysis comparing the mean fold-changes of both methods (x-axis) and the difference between them (y-axis). This plot allows the identification of any systematic difference between methods and possible outliers. Each circle represents an HSP gene and their colours the subtype for which the fold-change was calculated. The blue dotted line represents the mean difference between both techniques (0.02) and the light blue dashed line depicts the upper (0.88) and lower (− 0.84) limits of the 95% confidence interval of the differences. (PDF 175 kb)

Additional file 7: HSPs differential gene expression between tumour tissues. The values were determined by EdgeR ANOVA-like method performed on 20,531 genes from BRCA TCGA. Only HSPs values are showed. Each column includes log_2_ fold change values for all comparison, log_2_ mean counts per million (logCPM), F-statistic and corresponding *p*-values and FDR values. The conditions compared are Luminal A vs. Luminal B, Luminal A vs. HER2, Luminal A vs. Basal-like, Luminal B vs. HER2, Luminal B vs. Basal-like and HER2 vs. Basal-like. Comparison between HSP-Clusts were also considered, namely HSP-Clust I vs. HSP-Clust II, HSP-Clust I vs HSP-Clust III and HSP-Clust II vs. HSP-Clust III. (XLSX 35 kb)

Additional file 8: Dendrogram analysis of hierarchical clustering based on HSPs gene expression. The separation distance between branches was determined by silhouette technique. The highest coefficient corresponds to the optimal number of cluster, in this case k = 3. (PDF 99 kb)

Additional file 9: Summary of HSP subfamily Fold Change trends across PAM50 subtypes. Boxplot representing HSP subfamilies log_2_ fold change ranges by EdgeR method in the different molecular subtypes of breast cancer. (PDF 111 kb)

Additional file 10: Differential gene expression in BRCA TCGA tumours. Summary of EdgeR ANOVA-like differential gene expression showing the HSPs pairwise differences between tumour subtypes. Genes were grouped according to their corresponding families. Chaperonins were divided into three different types (type I, type II and BBs chaperonins), HSPH were distinguished from the rest of the HSP70 family and DNAJ were divided into their three subfamilies (A, B and C). The vertical blue lines represents baseline level from the reference subtype while the light blue points shows the fold change of the HSP genes in each pairwise comparison. Red dots are depicted for genes that had absolute log_2_ fold changes greater than 2. A) Shows the comparison between PAM50 molecular subtypes, and B) shows differences between HSP-Clust subtypes. (PDF 202 kb)

Additional file 11: HSP clusters characterization. A) Centroid of HSP clusters expression profiles for TCGA, METABRIC training and test set. The colour of the boxes in regard to the central dashed line represents down (blue) or upregulation (red) of the gene in the corresponding cluster. The continuous black line represents the mean expression values of each gene in the cluster compared to the mean of the same gene over all samples. B) Agreement between PAM50 and HSP clusters for METABRIC training and test sets. The size of the bars is in proportion to the number of samples in each category. C) Overall survival of HSP clusters for METABRIC training and test sets. Kaplan-Meier curves corresponding to HSP-Clust I, HSP-Clust II and HSP-Clust III. Statistical significance was evaluated by Log-Rank test. (PDF 214 kb)

Additional file 12: PAM50 subtypes overall survival in TCGA and METABRIC cohorts. (PDF 166 kb)

## Abbreviations

BRCA: Breast cancer
DEG: Differential expressed gene
ER: Oestrogen receptor
ERSR: Endoplasmic reticulum stress response
FDR: False discovery rate
HER2: HER2-enriched
HSP: Heat shock proteins
METABRIC: Molecular Taxonomy of Breast Cancer International Consortium
PR: Progesterone receptor
TCGA: The Cancer Genome Atlas
