# Supplementary figures and images for: Comprehensive transcriptomic analysis of heat shock proteins in the molecular subtypes of human breast cancer
Source: BMC Cancer. 2018 Jun 28;18:700. doi: 10.1186/s12885-018-4621-1 (PMC6022707; doi:10.1186/s12885-018-4621-1)

## Additional file 2: Data analysis workflow

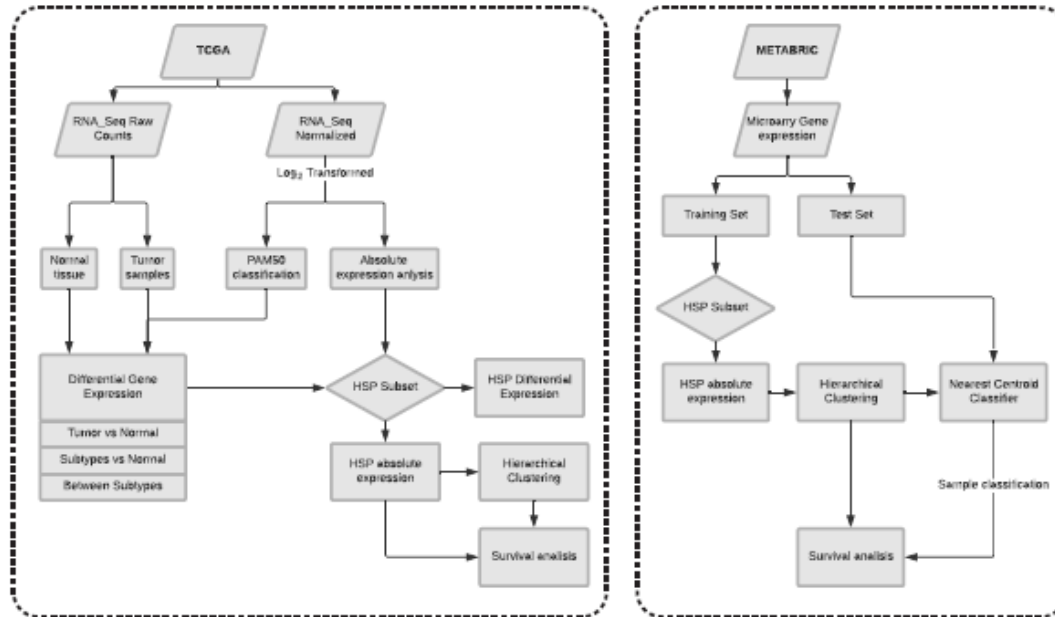

Supplement: Supplementary file 2 — Data analysis workflow. Schematic representation of HSPs transcriptomic and survival analysis process. (PDF 108 kb) [file 12885_2018_4621_MOESM2_ESM.pdf]

Additional file 3: PAM50 classification quality control of TCGA's samples I.

A

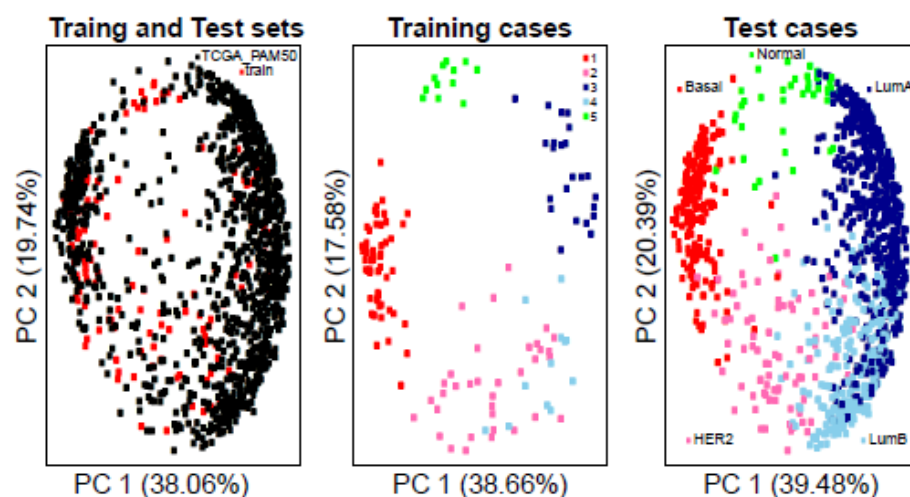

B

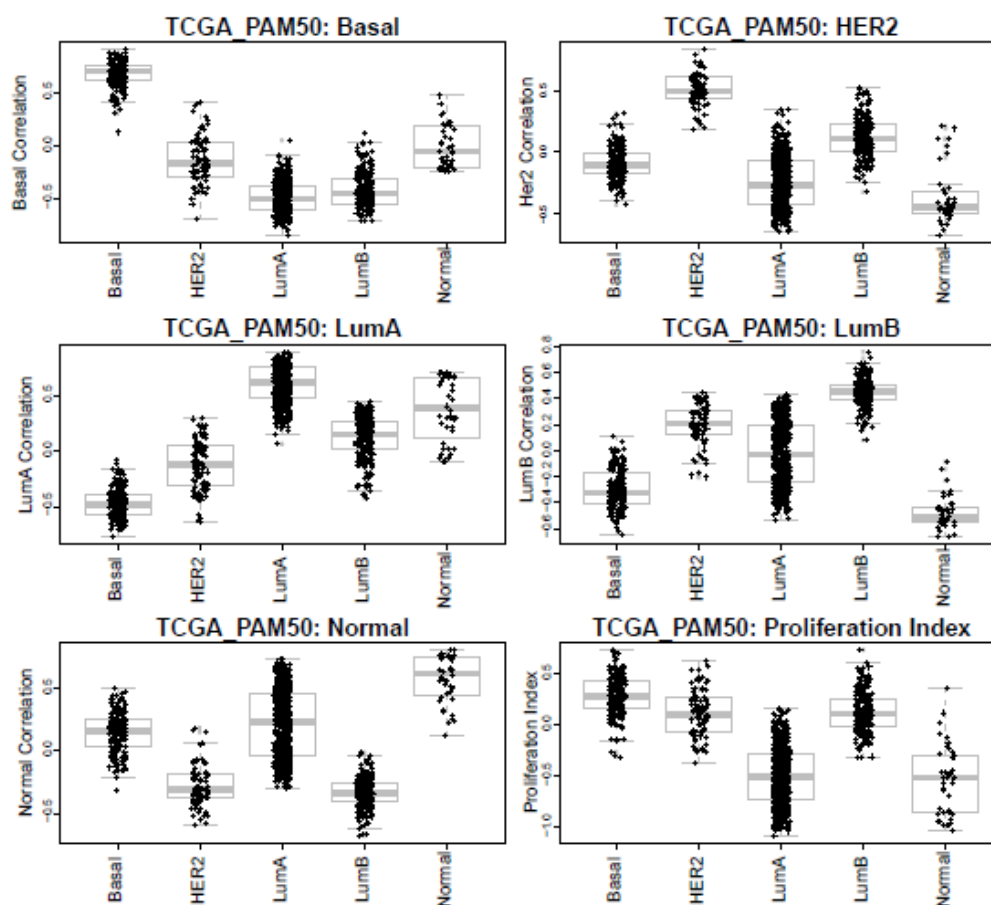

Supplement: Supplementary file 3 — PAM50 classification quality control of TCGA’s samples I. A) Principal components analysis of the training and test sets. Note the subtype clustering and the superposition between both datasets. B) Correlations between subtype assigned and the corresponding subtype centroids per sample and relation between subtypes and proliferation index. Each dot represents a single sample. (PDF 166 kb) [file 12885_2018_4621_MOESM3_ESM.pdf]

Additional file 4: PAM50 classification quality control of TCGA's samples II.

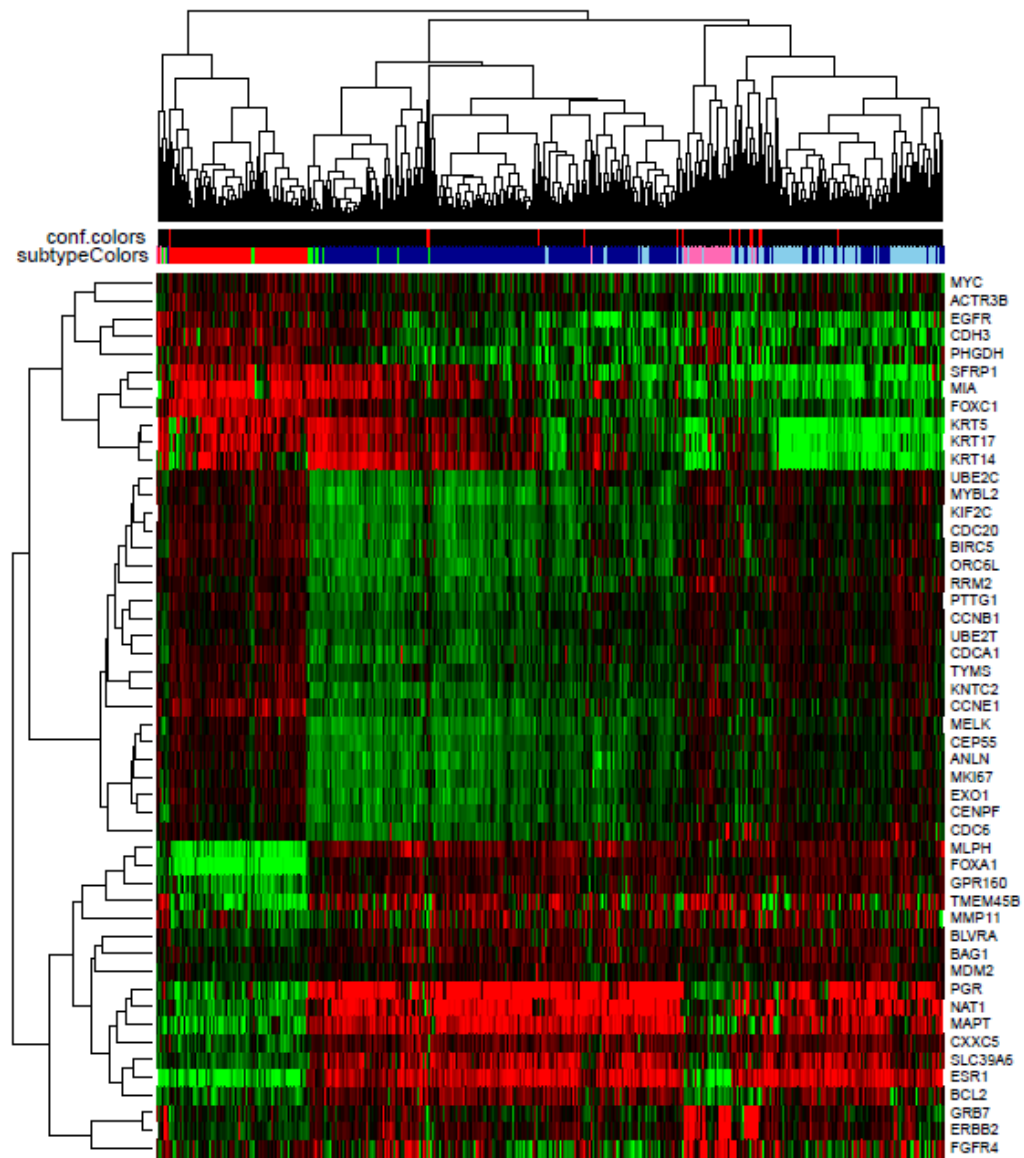

Supplement: Supplementary file 4 — PAM50 classification quality control of TCGA’s samples II. Unsupervised hierarchical clustering of samples according to PAM50 gene set expression. Note the consistency between the subtype assigned to each sample by PAM50 algorithm and the group composition determined by the clustering technique. (PDF 133 kb) [file 12885_2018_4621_MOESM4_ESM.pdf]

Additional file 6: Fold-change consistency between EdgeR and DESeq2 methods.

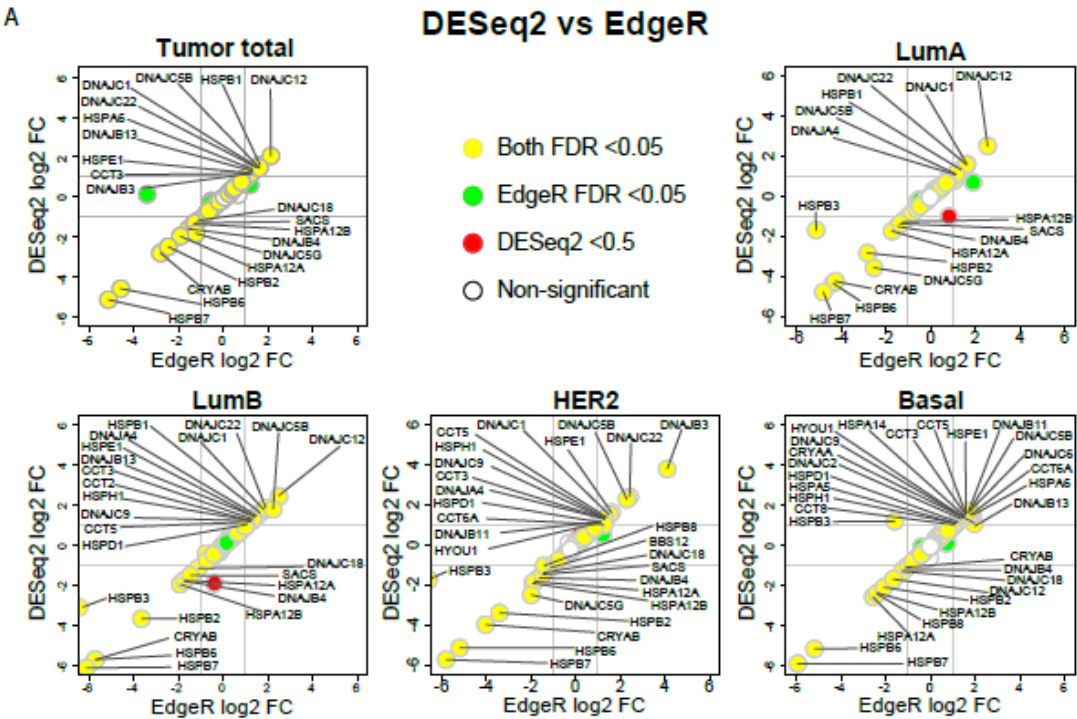

**B**

**Bland Altman Plot**

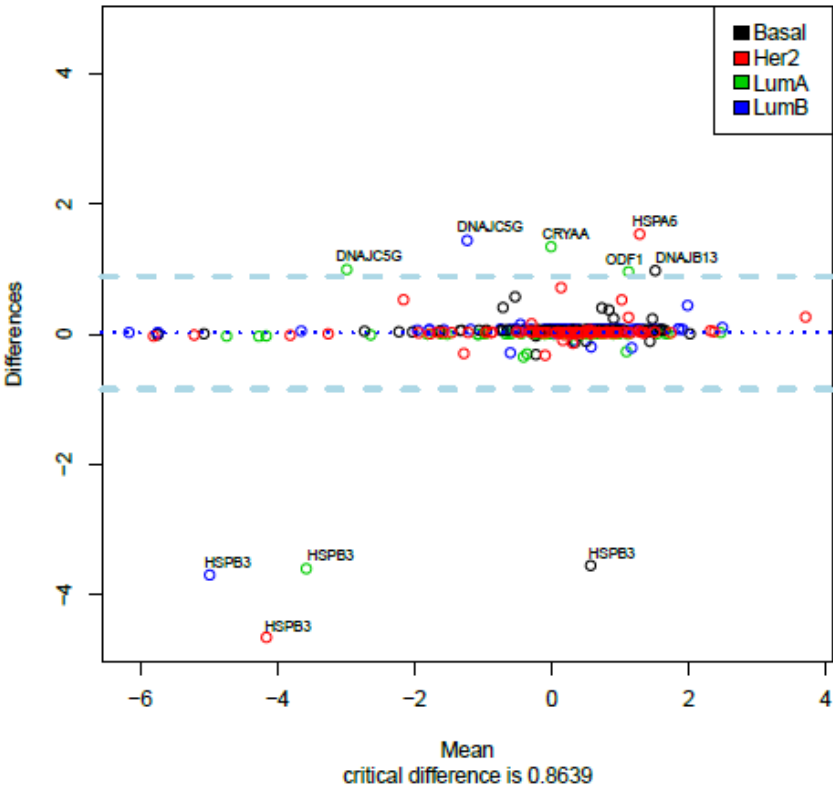

Supplement: Supplementary file 6 — Fold-change consistency between EdgeR and DESeq2 methods. A) Correlation analysis between fold-change obtained by both methods. The figure shows a tight linear trend between EdgeR and DESeq2 fold-change estimations. Genes found significant for both methods are represented in yellow circles, in green and red are genes significantly differentially expressed by one of the two methods and in white, genes with no significant changes by both techniques. B) Bland Altman analysis comparing the mean fold-changes of both methods (x-axis) and the difference between them (y-axis). This plot allows the identification of any systematic difference between methods and possible outliers. Each circle represents an HSP gene and their colours the subtype for which the fold-change was calculated. The blue dotted line represents the mean difference between both techniques (0.02) and the light blue dashed line depicts the upper (0.88) and lower (− 0.84) limits of the 95% confidence interval of the differences. (PDF 175 kb) [file 12885_2018_4621_MOESM6_ESM.pdf]

Additional file 9: Summary of HSP subfamily Fold Change trends across PAM50 subtypes

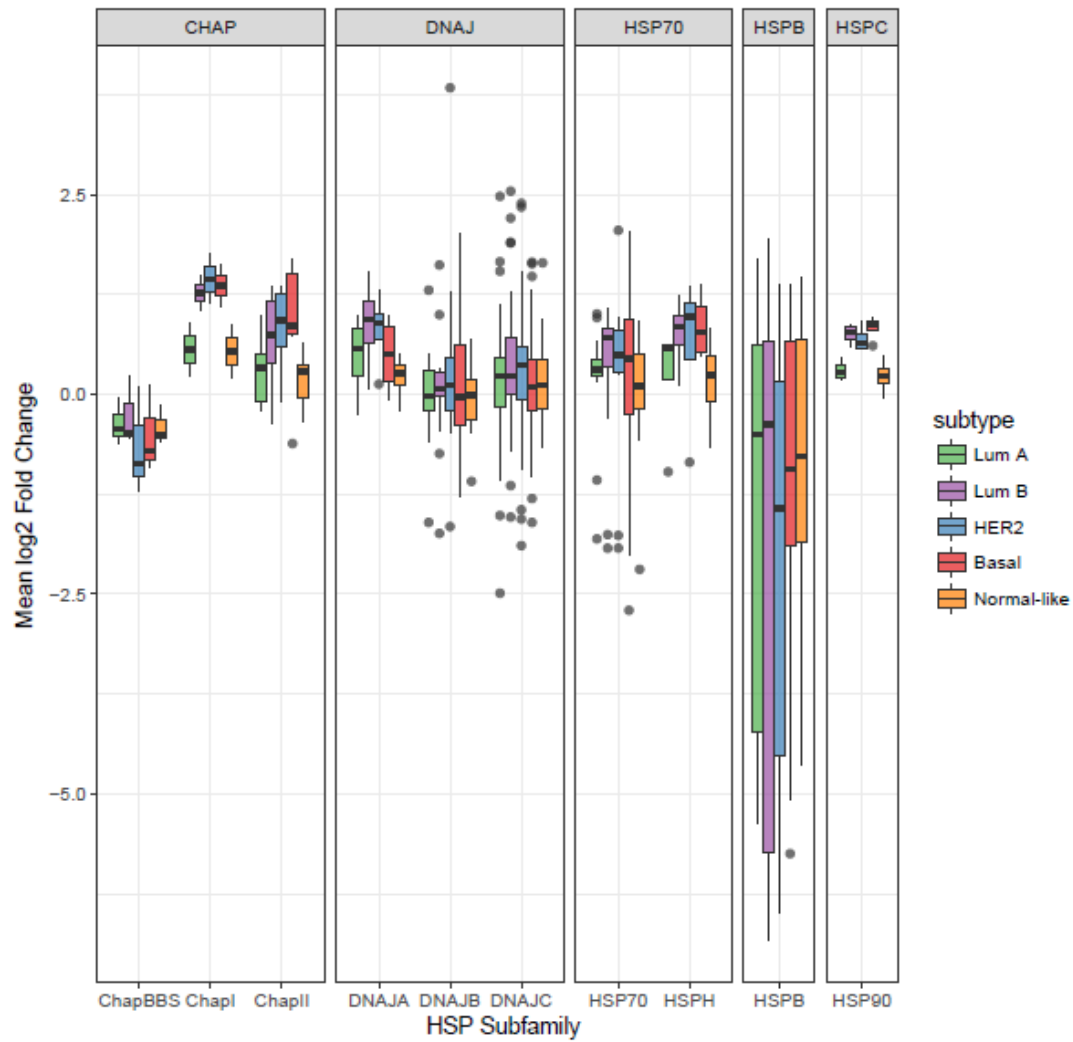

Supplement: Supplementary file 9 — Summary of HSP subfamily Fold Change trends across PAM50 subtypes. Boxplot representing HSP subfamilies log2 fold change ranges by EdgeR method in the different molecular subtypes of breast cancer. (PDF 111 kb) [file 12885_2018_4621_MOESM9_ESM.pdf]
