# Supplementary material for: Comprehensive transcriptomic analysis of heat shock proteins in the molecular subtypes of human breast cancer
Source: BMC Cancer. 2018 Jun 28;18:700. doi: 10.1186/s12885-018-4621-1 (PMC6022707; doi:10.1186/s12885-018-4621-1)

Additional file 8: Dendrogram analysis of hierarchical clustering based on HSP gene expression

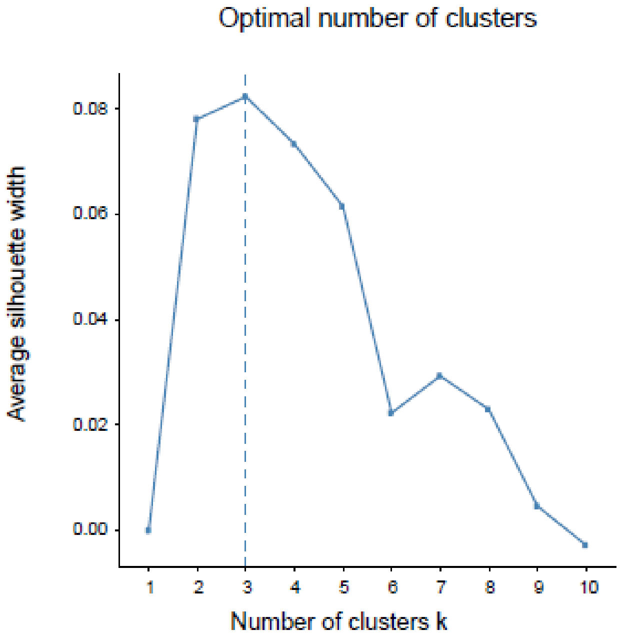

Supplement: Supplementary file 8 — Dendrogram analysis of hierarchical clustering based on HSPs gene expression. The separation distance between branches was determined by silhouette technique. The highest coefficient corresponds to the optimal number of cluster, in this case k = 3. (PDF 99 kb) [file 12885_2018_4621_MOESM8_ESM.pdf]
