# Supplementary material for: Comprehensive transcriptomic analysis of heat shock proteins in the molecular subtypes of human breast cancer
Source: BMC Cancer. 2018 Jun 28;18:700. doi: 10.1186/s12885-018-4621-1 (PMC6022707; doi:10.1186/s12885-018-4621-1)

Additional file 10 A: TCGA differential HSP gene expression between PAM50 breast cancer subtypes

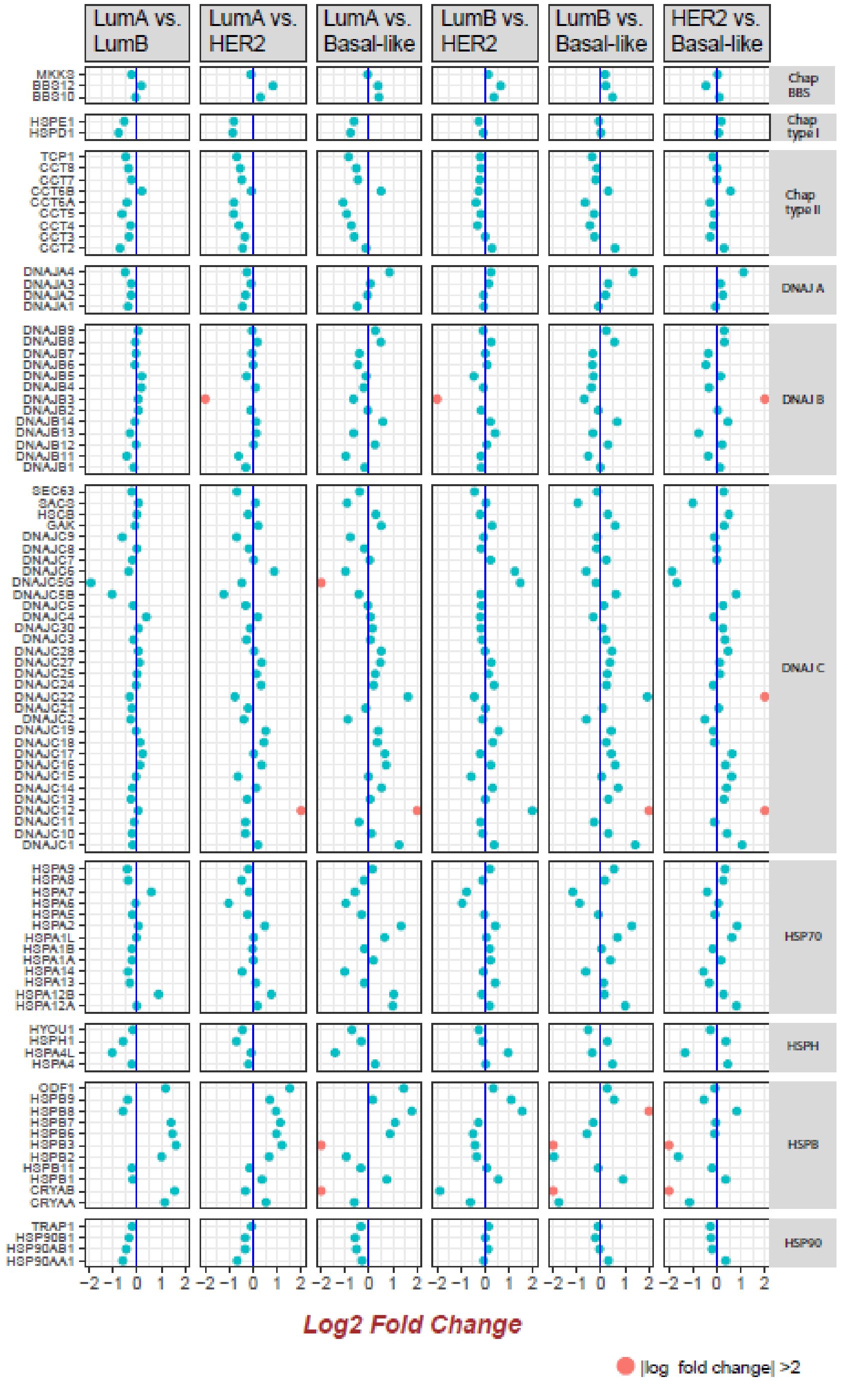

Additional file 10 B: TCGA differential HSP gene expression between HSP-Clusts

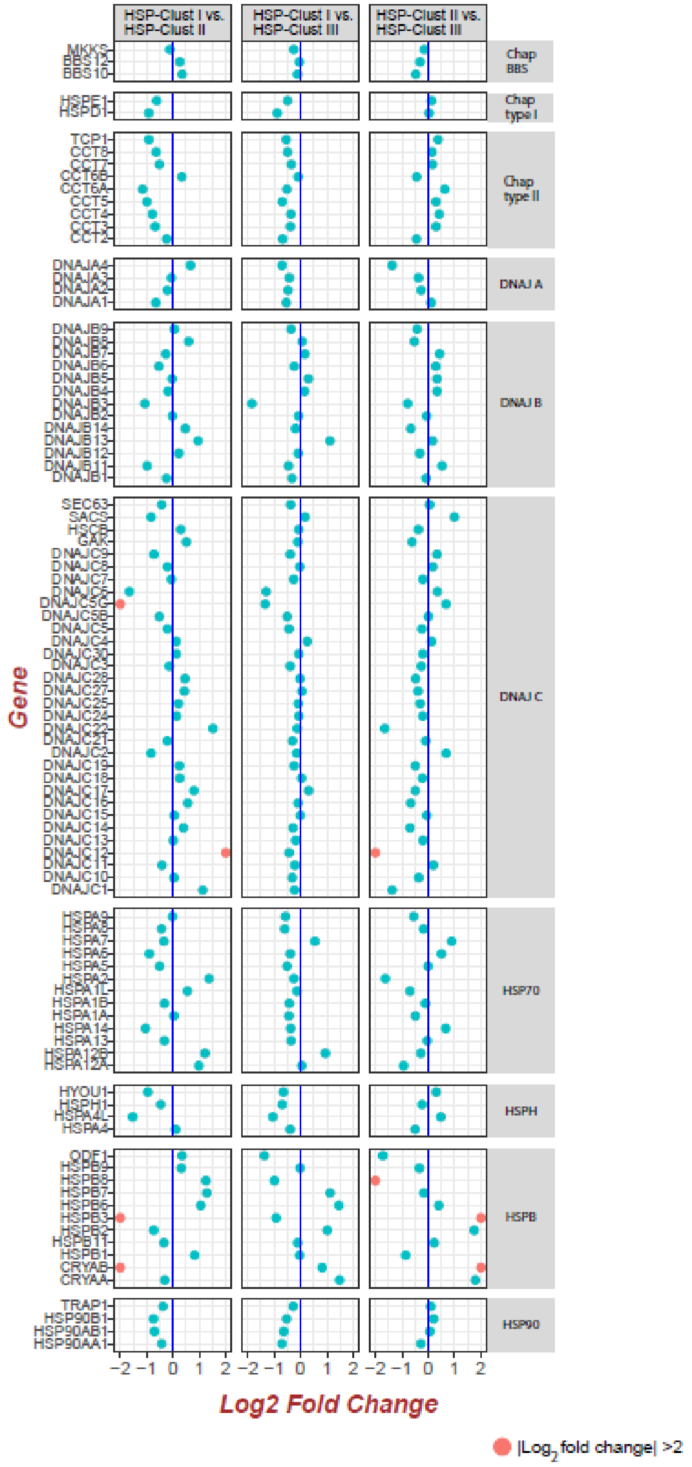

Supplement: Supplementary file 10 — Differential gene expression in BRCA TCGA tumours. Summary of EdgeR ANOVA-like differential gene expression showing the HSPs pairwise differences between tumour subtypes. Genes were grouped according to their corresponding families. Chaperonins were divided into three different types (type I, type II and BBs chaperonins), HSPH were distinguished from the rest of the HSP70 family and DNAJ were divided into their three subfamilies (A, B and C). The vertical blue lines represents baseline level from the reference subtype while the light blue points shows the fold change of the HSP genes in each pairwise comparison. Red dots are depicted for genes that had absolute log2 fold changes greater than 2. A) Shows the comparison between PAM50 molecular subtypes, and B) shows differences between HSP-Clust subtypes. (PDF 202 kb) [file 12885_2018_4621_MOESM10_ESM.pdf]
