# Supplementary material for: Comprehensive transcriptomic analysis of heat shock proteins in the molecular subtypes of human breast cancer
Source: BMC Cancer. 2018 Jun 28;18:700. doi: 10.1186/s12885-018-4621-1 (PMC6022707; doi:10.1186/s12885-018-4621-1)

Additional file 11: METABRIC-HSP Nearest centroid classifier and survival analysis

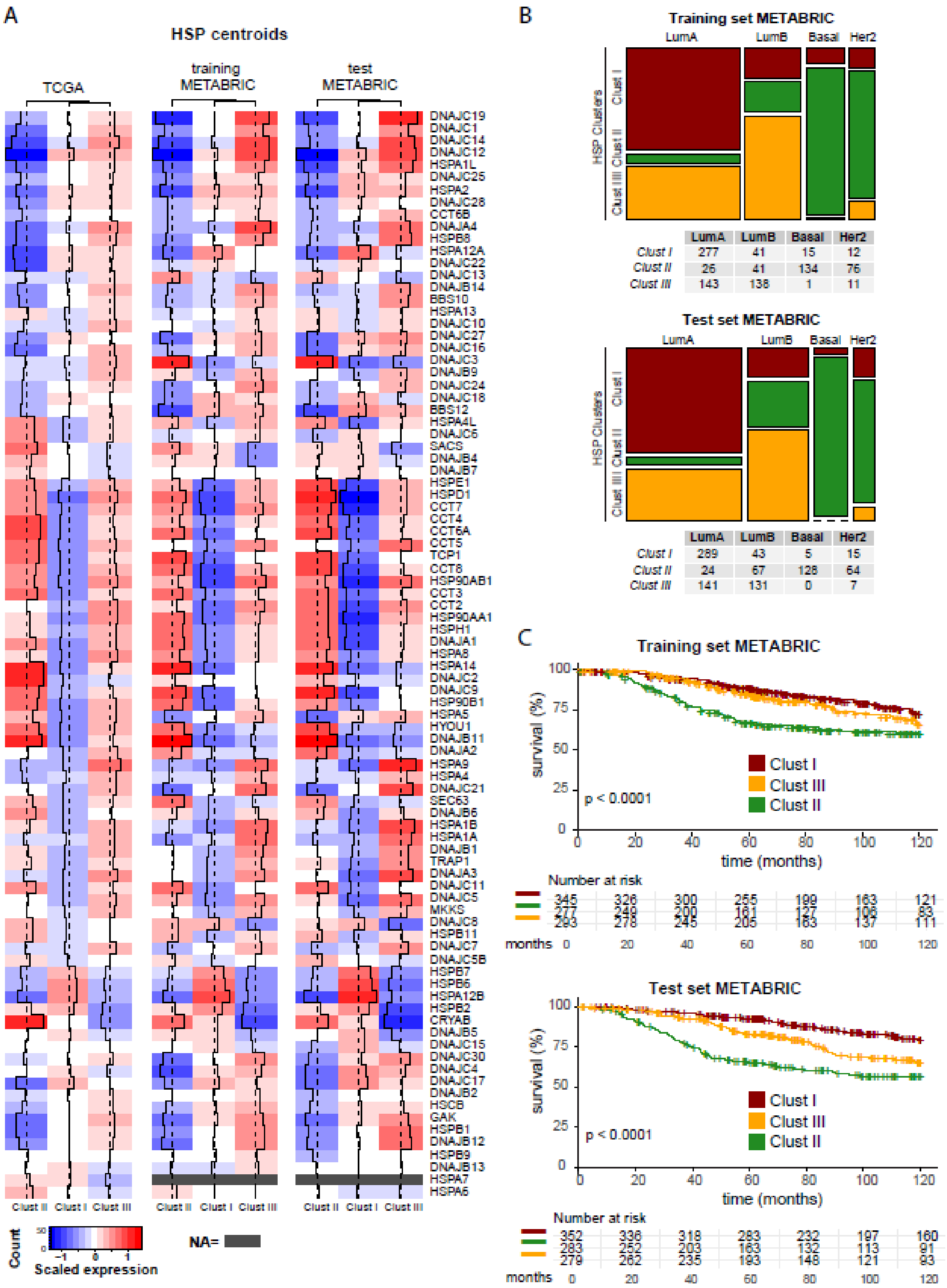

Additional file 10 B: TCGA differential HSP gene expression between HSP-Clusts

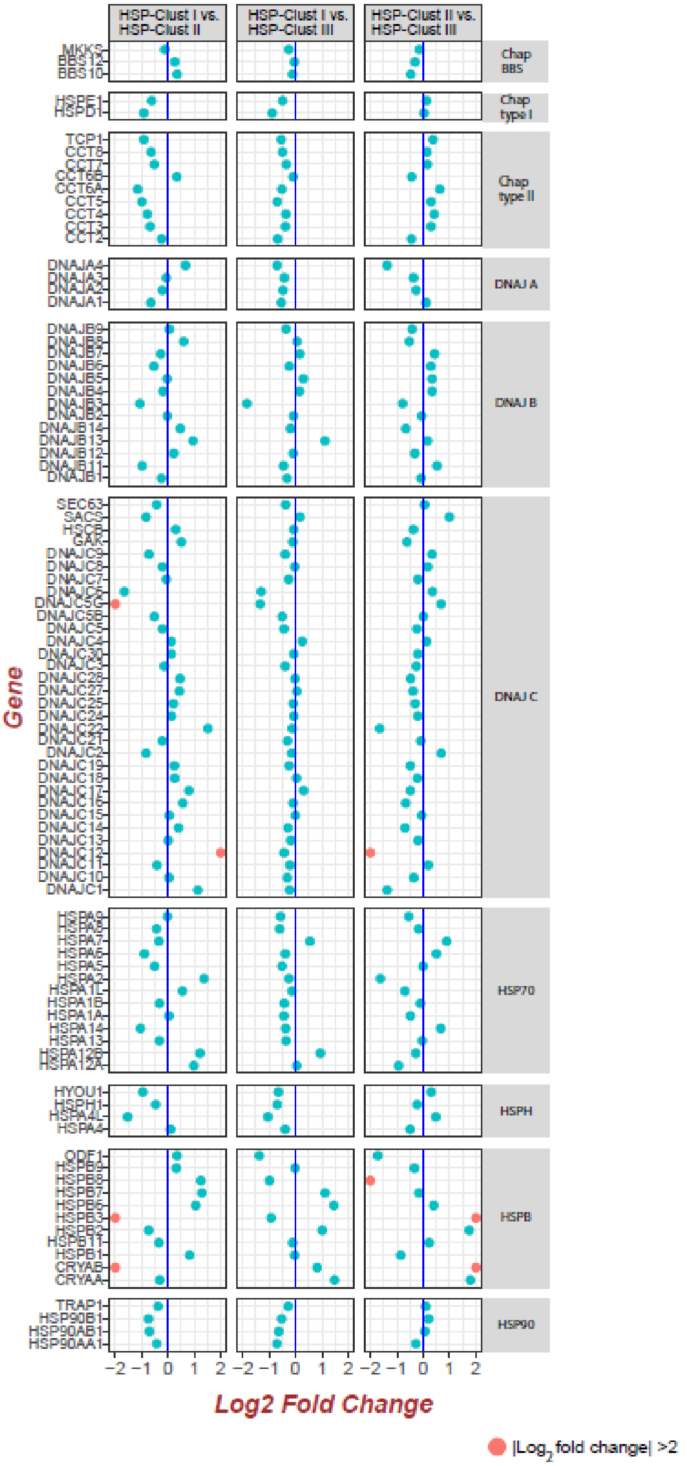

Supplement: Supplementary file 11 — HSP clusters characterization. A) Centroid of HSP clusters expression profiles for TCGA, METABRIC training and test set. The colour of the boxes in regard to the central dashed line represents down (blue) or upregulation (red) of the gene in the corresponding cluster. The continuous black line represents the mean expression values of each gene in the cluster compared to the mean of the same gene over all samples. B) Agreement between PAM50 and HSP clusters for METABRIC training and test sets. The size of the bars is in proportion to the number of samples in each category. C) Overall survival of HSP clusters for METABRIC training and test sets. Kaplan-Meier curves corresponding to HSP-Clust I, HSP-Clust II and HSP-Clust III. Statistical significance was evaluated by Log-Rank test. (PDF 214 kb) [file 12885_2018_4621_MOESM11_ESM.pdf]
