# Supplementary material for: Comprehensive transcriptomic analysis of heat shock proteins in the molecular subtypes of human breast cancer
Source: BMC Cancer. 2018 Jun 28;18:700. doi: 10.1186/s12885-018-4621-1 (PMC6022707; doi:10.1186/s12885-018-4621-1)

Additional file 12: PAM50 subtypes overall survival in TCGA and METABRIC cohorts

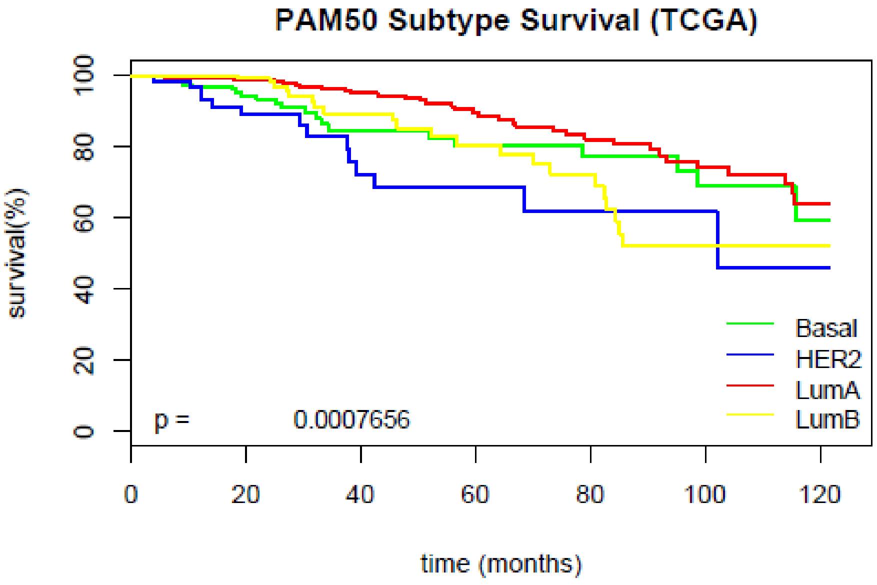

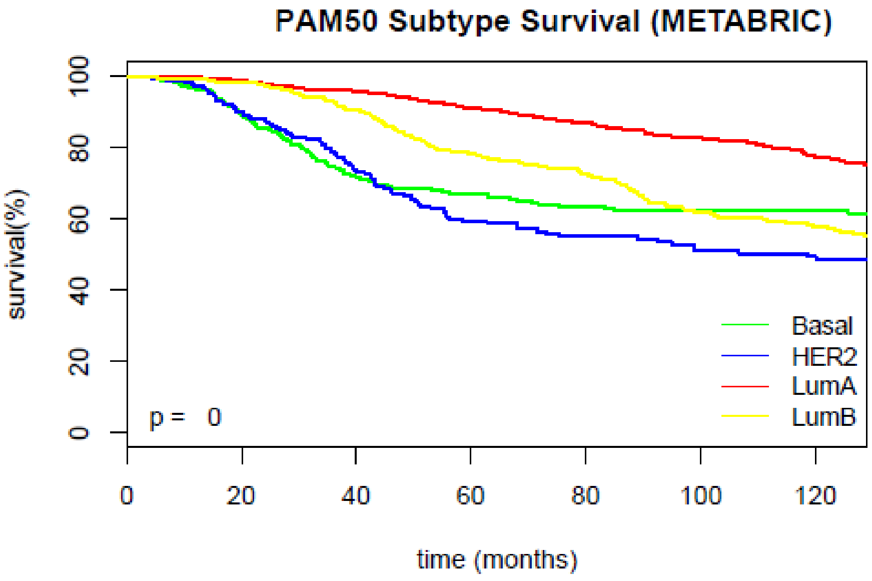

Additional file 10 B: TCGA differential HSP gene expression between HSP-Clusts

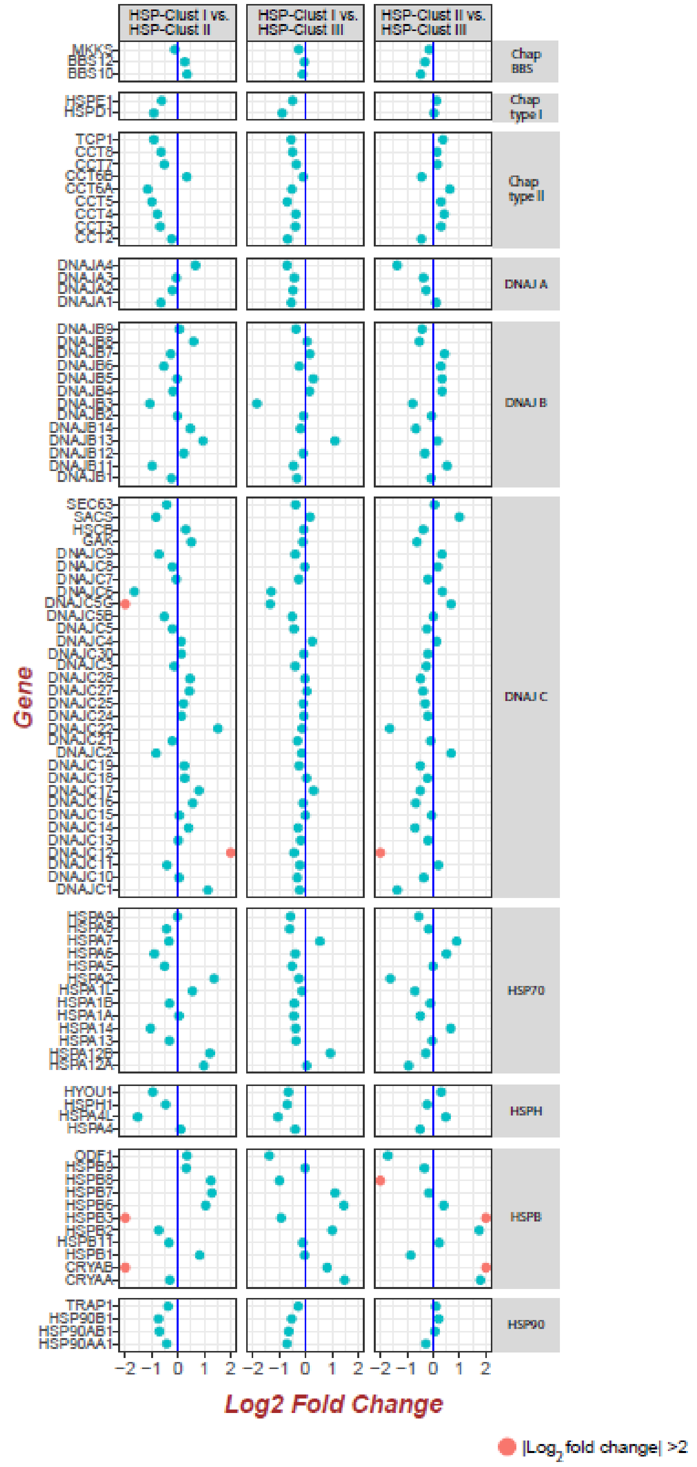

Supplement: Supplementary file 12 — PAM50 subtypes overall survival in TCGA and METABRIC cohorts. (PDF 166 kb) [file 12885_2018_4621_MOESM12_ESM.pdf]
